# Differential Transcriptomic Signatures of Small Airway Cell Cultures Derived from IPF and COVID-19-Induced Exacerbation of Interstitial Lung Disease

**DOI:** 10.3390/cells12202501

**Published:** 2023-10-21

**Authors:** Katie Uhl, Shreya Paithankar, Dmitry Leshchiner, Tara E. Jager, Mohamed Abdelgied, Bhavna Dixit, Raya Marashdeh, Dewen Luo-Li, Kaylie Tripp, Angela M. Peraino, Maximiliano Tamae Kakazu, Cameron Lawson, Dave W. Chesla, Ningzhi Luo-Li, Edward T. Murphy, Jeremy Prokop, Bin Chen, Reda E. Girgis, Xiaopeng Li

**Affiliations:** 1 Department of Pediatrics and Human Development, College of Human Medicine, Michigan State University, Grand Rapids, MI 49503, USAleshchi4@msu.edu (D.L.); abdelgi1@msu.edu (M.A.); dixitbh1@msu.edu (B.D.); raya.wael@yahoo.com (R.M.); jeremy.prokop@corewellhealth.org (J.P.);; 2 Corewell Health Medical Group, Grand Rapids, MI 49503, USAangela.peraino@spectrumhealth.org (A.M.P.); maximiliano.tamaekakazu@spectrumhealth.org (M.T.K.);; 3 Richard DeVos Lung Transplant Program, Corewell Health, Grand Rapids, MI 49503, USA; 4 Department of Pharmacology and Toxicology, Michigan State University, East Lansing, MI 48824, USA

**Keywords:** interstitial lung disease, ILD, idiopathic pulmonary fibrosis, IPF, COVID-19, coronavirus, RNA-sequencing, TGF-β1

## Abstract

Idiopathic pulmonary fibrosis (IPF) is a pathological condition wherein lung injury precipitates the deposition of scar tissue, ultimately leading to a decline in pulmonary function. Existing research indicates a notable exacerbation in the clinical prognosis of IPF patients following infection with COVID-19. This investigation employed bulk RNA-sequencing methodologies to describe the transcriptomic profiles of small airway cell cultures derived from IPF and post-COVID fibrosis patients. Differential gene expression analysis unveiled heightened activation of pathways associated with microtubule assembly and interferon signaling in IPF cell cultures. Conversely, post-COVID fibrosis cell cultures exhibited distinctive characteristics, including the upregulation of pathways linked to extracellular matrix remodeling, immune system response, and TGF-β1 signaling. Notably, BMP signaling levels were elevated in cell cultures derived from IPF patients compared to non-IPF control and post-COVID fibrosis samples. These findings underscore the molecular distinctions between IPF and post-COVID fibrosis, particularly in the context of signaling pathways associated with each condition. A better understanding of the underlying molecular mechanisms holds the promise of identifying potential therapeutic targets for future interventions in these diseases.

## 1. Introduction

Interstitial lung disease (ILD) is an umbrella term for a group of diseases characterized by lung fibrosis. The etiology driving idiopathic pulmonary fibrosis (IPF), a disease characterized by the accumulation of lung scar tissue, has so far remained undiscovered [1]. Approximately 50,000 people are diagnosed with IPF annually, and 40,000 patients die within the same time frame [2]. Injury to the lung induces fibrotic tissue to form, eventually inhibiting the lung’s ability to exchange oxygen. Structural changes observed in IPF patients include widening of the airways (known as traction bronchiectasis), heterogenous reticulation, and the formation of honeycomb cysts [1]. Recent evidence has suggested that small airways (those having a diameter of less than 2 mm) play an essential part in the pathogenesis of IPF [1,3,4,5]. The loss of small airways in chronic obstructive pulmonary disease (COPD) is well documented, but the investigation into the role of small airways in IPF remains limited [1,6]. One study found that patients suffering from IPF demonstrate a significant decrease in the number of terminal airways in the lung, even in minimally fibrotic regions [1]. A study performed in 2020 by Verleden et al. found that the small airways of IPF patients demonstrated distortion of the airways’ cellular lining and thickening of the airway walls due to fibrosis [4]. In the case of late-stage fibrosis, the dilated lumens in the small airways eventually lead to the formation of honeycomb cysts [6]. Authors of a 2021 study examining the early pathological features of IPF in whole explant lungs hypothesized that the loss of terminal bronchioles may precede the activation of the fibrotic response [6,7].

The novel severe acute respiratory syndrome coronavirus 2 (SARS-CoV-2, also known as COVID-19) that emerged in 2019 became characterized by respiratory illness and injury amongst humans. Patients diagnosed with COVID-19 experience mild flu-like symptoms that can progress into a more severe disease manifestation characterized by alveolar damage, epithelial injury, and pulmonary fibrosis [8]. A previous study on IPF patient lungs and fibrotic lesions from post-COVID-19 patient lungs found numerous mechanistic similarities, including increased extracellular matrix proteins, elevated levels of fibrotic and inflammatory cytokines, and reduced lung function [9,10]. A study published in 2022 found that patients diagnosed with COVID-19 had significantly higher instances of air trapping than healthy controls, providing evidence that small airway disease is present in COVID-19 patients [11]. Pulmonary fibrosis patients are at a higher risk of developing severe COVID-19 infection-related complications, and studies have shown that acute lung injury can worsen IPF [12,13].

Transforming growth factor beta (TGF-β1) is a pro-fibrotic cytokine whose expression is increased in human IPF lungs and bleomycin-induced fibrosis in animal models [5,14,15]. High levels of TGF-β1 were identified in acute-phase COVID-19 as well [16]. The cellular sources for the upregulation of the three isoforms of TGF-β (of which TGF-β1 is the most prevalent isoform found in pulmonary fibrosis) are the bronchial epithelium, myofibroblasts, alveolar macrophages, eosinophils, and hyperplastic type II alveolar epithelial cells (AECs) [14]. The binding of TGF-β1 to its receptor leads to the recruitment of the SMAD2 and SMAD3 transcription factors. When these factors are phosphorylated, they form a complex with SMAD4 and move into the nucleus. This complex interacts with other transcription factors in a signaling cascade that eventually leads to the cellular characteristics of fibrosis, such as collagen deposition, fibroblast accumulation, immune system activation, and inflammatory signaling. In the current study, cells were cultured at the air-liquid interface and treated with TGF-β1 to simulate the native fibrotic environment.

This research employed bulk RNA-sequencing on small airway cell cultures derived from patients to elucidate the transcriptomic signatures associated with IPF and the exacerbation of ILD induced by COVID-19, herein referred to as “post-COVID fibrosis”. TGF-β1 was introduced and compared against untreated control cultures, denoted as “baseline” conditions in a targeted subset of cultures. Given the shared structural characteristics between IPF and post-COVID fibrosis-afflicted lungs, a comparative analysis of the bulk RNA-sequencing data of small airway cells from both conditions was conducted. Furthermore, the impact of TGF-β1 treatment was examined across the three groups, and immunohistochemical staining was performed to elucidate potential rationales for observed variations in response to TGF-β1 between the two diseases. The comprehensive exploration of the influence of COVID-19 infection on the progression of pulmonary fibrosis holds the potential to unveil valuable targets for future therapeutic interventions.

## 2. Materials and Methods

### 2.1. Human Lung Explant Samples

A total of 18 lung explant samples were obtained from Corewell Health, consisting of 10 IPF patients and 4 post-COVID fibrosis patients undergoing lung transplantation (Supplementary Table S1). All samples were from male patients, except for one non-IPF control from a female lobectomy patient. The average age at transplantation was 62.9 years. Three donors from each category were used to generate cell cultures for RNA sequencing. Before transplantation, patients met IPF diagnostic criteria, including decreased lung volumes, dyspnea, and hypoxemia [17]. A pathological assessment confirmed fibrosis, which was visually observed in small airway tissue samples taken from the lobe’s distal end. Post-COVID fibrosis patients had pre-existing pulmonary fibrosis exacerbated by COVID-19. All patients in both groups had pulmonary fibrosis before transplantation.

**Figure 1:**
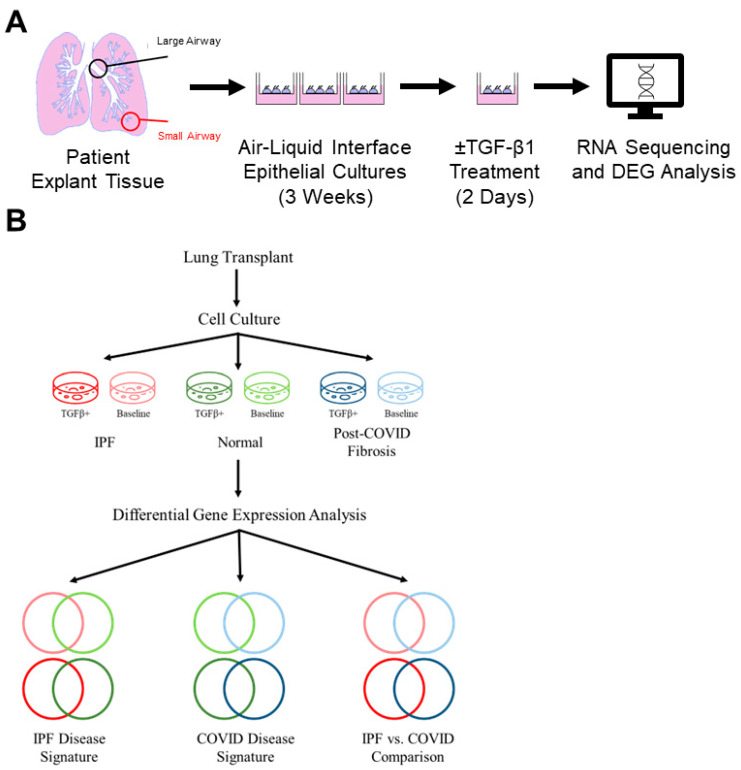
Culturing of small airway cells from patient lung tissues. (**A**) Simplified diagram of experimental workflow. Human small airway epithelial cells were harvested from the distal lobes of lung transplant patients. These cells were expanded and cultured onto transwell cell culture membranes. After 14 days, cultures were treated with TGFβ1 to mimic native fibrotic tissue conditions before being submitted for RNA sequencing. (**B**) Schematic depicting the collection and analysis of bulk RNA-sequencing data; “COVID” = post-COVID fibrosis, or pre-existing IPF exacerbated by COVID-19.

Donor lungs not suitable for transplantation were provided by Lung Bioengineering Inc. for immunohistochemical staining. Non-IPF control lung cell cultures were generated from healthy adjacent tissue in patient lobectomy samples for RNA-sequencing and RT-PCR. Lobectomy samples were chosen for ease of procurement and clinical data availability. “Normal” refers to lung samples without disease, and “untreated control” refers to cell cultures not treated with TGF-β1. Data collection and analysis details are in Figure 1B.

Clinical details for each patient, including pathology, smoking history, and COVID-19 infection time, are in Supplementary Table S1. Three donor lung samples used for IHC were from Lung Bioengineering, Inc., with limited donor data. All lobectomy patients and some IPF and post-COVID fibrosis patients had a smoking history. FEV1 and FVC predictions, ventilator use before transplantation, and COVID-19 infection details were included. Post-COVID fibrosis patients tested positive between May 2020 and October 2021, with an average of 249 days between infection and transplant. IPF and non-IPF controls did not test positive for COVID-19 before their respective procedures.

### 2.2. Isolation and Propagation of Distal Small Airways

The small airways were micro-dissected from the patient’s lungs and digested in a buffer containing protease from *Streptomyces griseus* (P5147, Millipore-Sigma, St. Louis, MO, USA), 1X Gentamicin (15750060, Gibco, Waltham, MA, USA), 1X penicillin/streptomycin (15140122, Gibco), and DNase I (10104159001, Millipore-Sigma, St. Louis, MO, USA). Epithelial cells were isolated and expanded by culturing with PneumaCult™-Ex Plus Medium (05040, STEMCELL Technologies, Vancouver, Canada) at 37 °C, 5% CO_2_, for approximately one week. PneumaCult™-Ex Plus Medium is a serum- and BPE-free medium formulated to expand human primary airway epithelial cells. The cells were cultured at the air-liquid interface (ALI) on transwell plates (3413, Corning, Corning, NY, USA), with or without TGF-β1, for three weeks before being collected for experimentation (Figure 1A). A period of at least 14 days is required for basal cells to differentiate into other cell types after the addition of TGFβ1 and for a fibrotic phenotype to emerge after treatment with adenoviral TGF-β1 [18]. For this study, TGF-β1 was added two days before harvesting. All IPF and post-COVID fibrosis cell cultures were generated from patients with a pathological diagnosis of usual interstitial pneumonia (UIP), also known as IPF.

### 2.3. RNA Isolation and Sequencing

The sample tissue was first homogenized in TRIzol™ Reagent (15596026, Invitrogen, Waltham, MA, USA) for RNA isolation and then treated with chloroform. The aqueous phase was added to an equal volume of 70% ethanol and transferred to a column from the RNeasy Mini Kit (74104, QIAGEN, Hilden, Germany). The protocol for RNA extraction was carried out according to the manufacturer’s instructions. RNA concentration and quality measurements were taken using a NanoDrop™ OneC Microvolume UV-Vis Spectrophotometer (840274200, Thermo Scientific, Waltham, MA, USA). RNA sequencing samples were submitted to the Genomics Core at the Van Andel Institute for bulk RNA sequencing with an Illumina NovaSeq 6000 instrument. The data were uploaded to the National Center for Biotechnology Information (NCBI) database (GSE225549).

### 2.4. Differential Expression Analysis

RNA-sequencing data was processed using the Toil RNA-seq Pipeline to calculate transcript abundance (BD2KGenomics, 2019, toil-rnaseq, (Version 4.0.3) https://github.com/BD2KGenomics/toil-rnaseq.git, accessed on 18 October 2023). The count data were submitted to DESeq2 and EdgeR for differential expression analysis using RStudio (2023.09.1+494, Boston, MA, USA [19,20]. Genes with a *p*-value ≤ 0.01 and |log 2 Fold Change| ≥ 1 were considered significant for all downstream analyses. The Ingenuity Pathway Analysis (IPA) tool from QIAGEN (QIAGEN Inc., (Version 101138820) https://digitalinsights.qiagen.com/IPA, accessed on 18 October 2023) was used to identify canonical pathways and upstream regulators represented by the list of differentially expressed genes [21]. Gene ontology analysis was performed using the Gene Ontology resource [22,23]. A list of common genes was determined by comparing the list of DEGs from the baseline condition and TGF-β1-treated cultures. The activation states and -log (*p*-Value) for the IPA canonical pathways and upstream regulators were predicted using the DEGs and associated fold change data from the baseline condition as input. Due to the large number of DEGs in each disease dataset, the transcriptomic signature was defined as the DEGs shared by both culture conditions. GenePattern 2.0 generated all heatmaps used for gene expression visualization [24].

### 2.5. cDNA Synthesis and Real-Time PCR

cDNA was synthesized from the isolated RNA samples using the SuperScript™ IV VILO™ Master Mix (11756050, Invitrogen, Waltham, MA, USA) with ezDNase enzyme treatment according to the manufacturer’s protocol. Individual master mixes for each gene of interest (*ACTB*, *BMP7*, *BMPR1A*, *BMPR1B*, and *FOXM1*) were prepared using the TB Green^®^ Premix Ex Taq II (TLi RNaseH Plus) reagent kit (RR820A, Takara Bio, San Jose, CA, USA) and primer sets ordered from Sigma-Aldrich (Supplementary Table S2). The reaction was carried out using the CFX96™ Real-Time System (Bio-Rad, Hercules, CA, USA) using the following settings: a holding stage of 30 s at 95 °C, followed by 40 cycles of 95 °C for 5 s and 60 °C for 30 s, and ending with the melt curve stage. The instrument software from Bio-Rad was used to quantify the gene expression levels based on Ct and normalized to the reference gene *ACTB*. Expression level comparisons were carried out using an unpaired *t*-test, and the data are expressed as the mean fold change ± SEM.

### 2.6. Protein Extraction and Western Blotting

Frozen small airway tissue isolated from donor lungs was homogenized in 1X RIPA lysis buffer (R0278, Millipore-Sigma, St. Louis, MO, USA) and protease inhibitors (A32955, Themo Scientific, Waltham, MA, USA). Protein samples were loaded onto a Bolt™ 4–12%, Bis-Tris, 1.0 mm, mini protein gel (NW04122BOX, Invitrogen, Waltham, MA, USA). An iBlot™ Transfer System (IB24001, Invitrogen, Waltham, MA, USA) was used to transfer the proteins onto a PVDF membrane. The membranes were blocked with 3% milk/TBST for 1 h, followed by an overnight incubation at 4°C with primary antibodies. Primary antibodies included SCGB3A2 (ab181853, Abcam, Waltham, MA, USA), SFTPA (AB3420, Millipore-Sigma, St. Louis, MO, USA), SMAD7 (MAB2029, R&D Systems, Minneapolis, MN, USA), β-actin (A2228, Millipore-Sigma, St. Louis, MO, USA), and GAPDH (14C10, Cell Signaling Technology). After washing with TBST, the membranes were incubated with anti-rabbit or anti-mouse HRP-coupled secondary antibodies for 2 h and imaged using the ChemiDoc™ MP Imaging System (BioRad, Hercules, CA, USA). Where applicable, chemiluminescent signals were quantified using FIJI and the data are expressed as the mean intensity fold change ± SEM [25].

### 2.7. Immunohistochemistry

#### 2.7.1. Immunostaining and Quantification of Patient-Derived Cell Cultures for Epithelial Cell Markers

Small airway cell cultures were generated from consented transplant patient distal lung tissue. Cell culture membranes were fixed with 4% paraformaldehyde, embedded in Scigen Tissue-Plus™ O.C.T. Compound (23-730-571, Fisher Scientific, Waltham, MA, USA), and mounted onto slides. Ciliated cells were detected by staining with mouse anti-acetylated α-tubulin (T7451, Millipore-Sigma, St. Louis, MO, USA) at a 1:1000 dilution. The slides were washed with TBST and then incubated with a 1:1000 dilution of anti-mouse secondary (A-11001, Invitrogen, Waltham, MA, USA). After another series of washes with TBST and ddH2O, coverslips were mounted using ProLong™ Diamond Antifade Mountant with DAPI (P36962, Molecular Probes/Invitrogen, Waltham, MA, USA). Coverslips were sealed using nail polish and allowed to sit for 24 h before being imaged at 40× with a Nikon C2 confocal microscope. The percentage of ciliated cells was determined by dividing the number of ciliated cells in the image by the total number of surface epithelial cells. Three fields were analyzed from three distinct donors for the non-IPF control and the IPF cell cultures for nine measurements for each category. For the post-COVID fibrosis cultures, samples from two donors were utilized, with three fields studied from one donor and four from the other. The principal investigator trained two observers on the criteria for identifying ciliated cells. All IPF and post-COVID fibrosis samples used for IHC were harvested from patients with pre-existing ILD.

#### 2.7.2. Immunofluorescent Detection of pSMAD1/5/8 in Patient Lung Tissue

Small airway tissue was isolated from the patient’s lungs and fixed in 4% paraformaldehyde (4% PFA) at 4 °C for at least 24 h. The tissue was then incubated in 15% sucrose, in 30% sucrose, and in a solution of 30% sucrose and 50% Scigen Tissue-Plus™ O.C.T. Compound (23-730-571, Fisher Scientific) for 24 h each. After the sucrose gradient was complete, the tissue was embedded in O.C.T. Slides were fixed in 4% PFA and permeabilized with 0.1% Triton X-100 (X100, Sigma-Aldrich). Sections were blocked with 5% BSA (A30075, Research Products International, Mount Prospect, IL, USA)/1% goat serum (G9023, Millipore-Sigma, St. Louis, MO, USA) in PBS, and then, sections were incubated overnight with rabbit anti-p-SMAD1/5/8 primary antibody (AB3848, Millipore-Sigma, St. Louis, MO, USA) at 4 °C. Sections were washed with TBST and then incubated with 1:1000 anti-rabbit secondary antibody (A-21069, Invitrogen, Waltham, MA, USA) in 2.5% BSA/0.5% goat serum in PBS for 1 h at room temperature. A series of washes with TBST and ddH2O was carried out, and coverslips were mounted to the slides using ProLong™ Diamond Antifade Mountant with DAPI (P36962, Molecular Probes/Invitrogen, Waltham, MA, USA). The slides were imaged using a Nikon C2 confocal microscope. The nuclear colocalization of pSMAD1/5/8 was determined by quantifying individual nuclei using the FIJI software (version 1.54f) [25]. Three fields per image in each disease category were quantified. Signal quantification was conducted using analysis software available from Nikon. GraphPad version 9.4.1 for Windows (GraphPad Software, Boston, Massachusetts USA, www.graphpad.com) was used to perform ordinary one-way ANOVA between groups; a *p*-value ≤ 0.01 was considered significant, and experimental data are expressed as the mean ± SEM.

## 3. Results

### 3.1. Patient-Derived Small Airway Cell Cultures Maintain Cell Markers and Characteristics Found in Fibrotic Lung Tissue

#### Patient-Derived Small Airway Cell Cultures Show Evidence of Ciliated Cells

**Figure 2:**
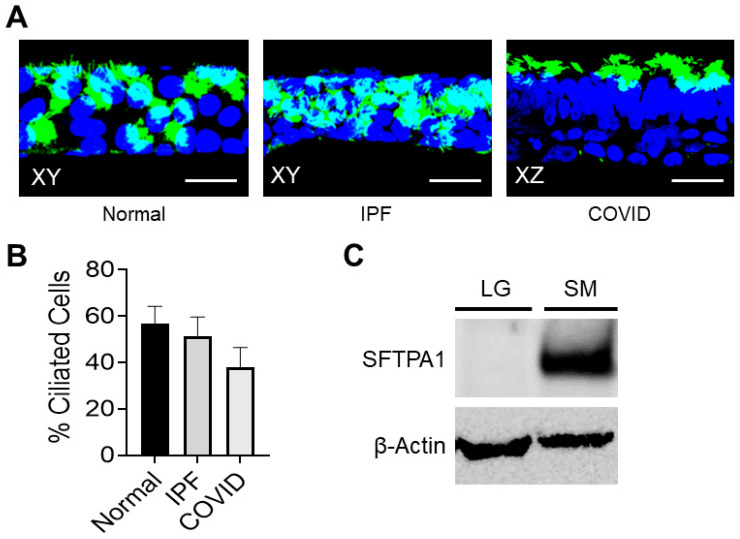
The detection of epithelial cell markers. (**A**) Immuno-staining of epithelial cells cultured on transwell membranes; ciliated cells (green) are present in the non-IPF control lung, IPF, and post-COVID fibrosis cell cultures. Images were taken at a magnification of 60×; scale bars represent 50 μM. (**B**) Percent of ciliated cells present in the surface epithelial cells of each sample type cultured on the transwell membranes. (**C**) Western blot showing the detection of the SFTPA protein in human large and small airway cultures from a pulmonary fibrosis patient.

Small airway cells taken from IPF patient lungs were expanded into cell cultures and stained for acetylated tubulin, a marker of ciliated cells (Figure 2A). The nature of the small airway hindered the isolation of primary cells from lung tissue; the authors found that the harvest and expansion of cells taken from distal lung tissue resulted in successful cultures of small airway epithelial cells. The staining results demonstrated that the small airway cell cultures were positive for ciliated cells (Figure 2A). The number of surface epithelial cells displaying cilia was quantified for each sample type. Membranes from the non-IPF control cell cultures had an average of approximately 56.9% ciliated surface epithelial cells. In comparison, the IPF and post-COVID fibrosis cell cultures had an average of 51.4% and 37.8%, respectively (Figure 2B). Protein from large and small diseased airways was extracted and analyzed using Western blotting techniques (Figure 2C). Surfactant protein A (SFTPA1), a protein with alveolar type II specificity, was detected only in the small airway cell culture lysate.

### 3.2. Determination of IPF Transcriptomic Signature

#### 3.2.1. RNA-Sequencing of IPF and Non-IPF Control Small Airway Cells Demonstrate Unique Transcriptomic Signatures

**Figure 3:**
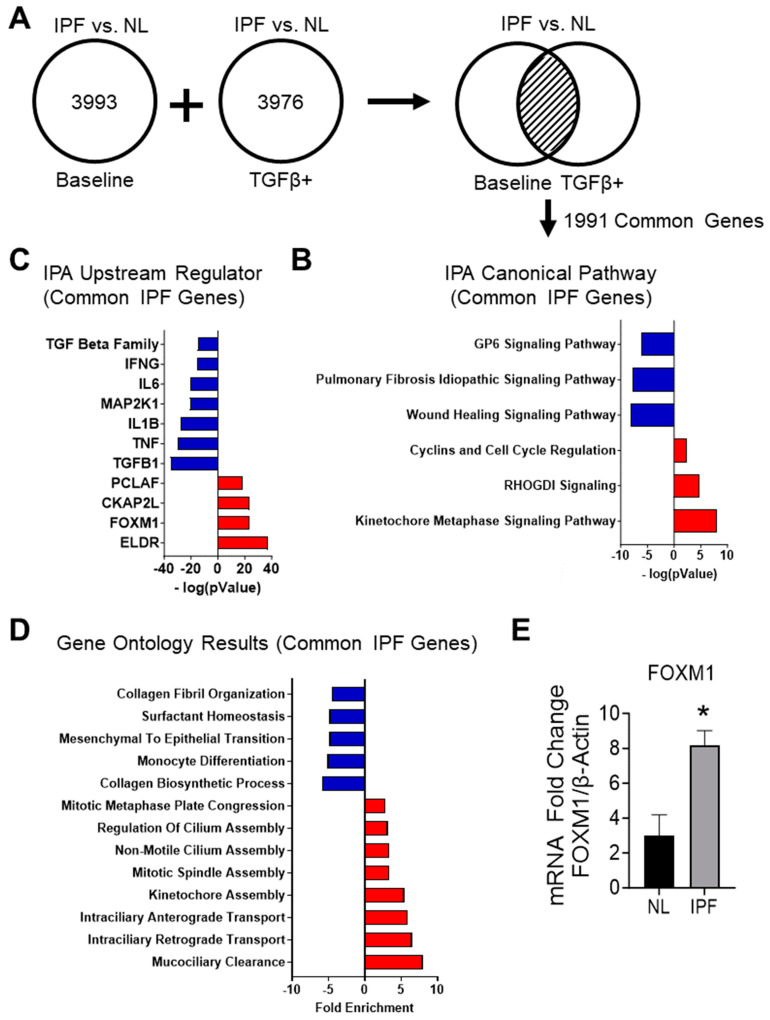
Analysis of the transcriptomic disease signature for small airway cells cultured from IPF patients. (**A**) Schematic showing the determination of common DEGs for the IPF vs. NL comparison. The transcriptomic signature was found by comparing the DEGs between IPF and non-IPF control cultures in both TGF-β1 treated and untreated culture conditions. The common genes were then used for downstream analyses. (**B**) IPA canonical pathway results for the common IPF cell culture genes relative to the non-IPF control samples. (**C**) IPA upstream regulator results for common IPF cell culture genes relative to the non-IPF control samples. (**D**) Gene ontology results of the common genes in the IPF small airway cell cultures. (**E**) RT-PCR results of *FOXM1* expression in the non-IPF control vs. IPF small airway cell cultures (*N* = 3, * indicates *p*-value < 0.05).

IPF cells were harvested from the distal lungs of IPF patients undergoing lung transplantation. Non-IPF lung small airway control cells were harvested from patients without airway disease. The transcriptomic signature of the IPF cell culture was determined by comparing the DEGs from the IPF vs. NL analyses under both culture conditions (Figure 3A). The baseline condition represents the condition of the cells as harvested from their native tissue; the addition of TGF-β1 was meant to stimulate the cell to activate fibrotic pathways that may have been switched off during the culturing process. The intersection of these two culture conditions was defined as the transcriptomic disease signature for this study; the baseline and the TGF-β1-treated culture condition shared 1991 DEGs (Figure 3A). These genes were used for downstream canonical pathways, upstream regulators, and gene ontology analyses.

#### 3.2.2. Analyzing the Canonical Pathways in Small Airway Cultures of IPF Compared to Non-IPF Controls Reveals a Suppression of Pathways Associated with IPF

The list of common DEGs for the IPF vs. non-IPF control small airway culture comparison was submitted as input to the Ingenuity Pathway Analysis (IPA) tool from QIAGEN. Genes and their associated fold changes were categorized based on QIAGEN’s database of pathways and regulators. The top pathway analysis results for the IPF vs. non-IPF control cell cultures shared DEGs included the kinetochore metaphase signaling pathway, the wound healing pathway, and the GP6 signaling pathway (Figure 3B) (Supplementary Table S3). The pathways predicted to be activated were the kinetochore metaphase signaling pathway, RHOGDI signaling, cyclins and cell cycle regulation, and EIF2 signaling. The pulmonary fibrosis idiopathic signaling and wound healing pathways were predicted to be inhibited in the IPF cell cultures compared to the non-IPF controls.

#### 3.2.3. Upstream Regulators Associated with Common IPF Genes Predict the Inhibition of the TGF-β1-Signaling Pathway and Host Immune Response

An essential function within IPA is the identification of significant upstream regulators and their predicted activation states. Data from the shared DEG list were used to identify upstream regulators and predict their activation states (Figure 3C and Supplementary Table S4). Among the top regulators expected to be activated were molecules involved in the cell cycle and the mediation of DNA damage, such as ELDR, FOXM1, and CKAP2L. Genes related to the host immune response and inflammation signaling (*TNF*, *IL-B*, and *IL-6*) were predicted to be inhibited. Although the experimental cultures were treated with TGF-β1 before sequencing, TGF-β1 signaling was the most significant upstream regulator in the IPF cell cultures compared to the non-IPF controls. It is worth noting that all TGF-β family members, including TGF-β2 and TGF-β3, were predicted to be inhibited, as represented by the TGF beta entry.

#### 3.2.4. Gene Ontology Results for Common IPF Genes Show Activation of Ciliary Movement and Cell Cycle Regulation and a Downregulation of Genes Associated with ECM Components and Immune Response

The gene ontology results for the upregulated DEGs associated with the IPF vs. non-IPF control cell culture comparison demonstrated a trend towards ontologies relating to the organization of cilia or cell cycle regulation (Figure 3D and Supplementary Table S5). The gene ontology with the most significant fold enrichment was mucociliary clearance, followed by anterograde and retrograde intraciliary transport; all are associated with ciliary movement. Ontologies corresponding to the cell cycle included kinetochore assembly, regulation of mitotic cytokinesis, and mitotic spindle assembly. The gene ontologies for the downregulated DEGs were more varied than the upregulated DEG results (Supplementary Table S6). In addition, immune-related entries, such as monocyte differentiation, reflected the trend seen in the inhibited upstream regulator results for the IPF cell cultures (compared to the non-IPF controls).

#### 3.2.5. Transcriptomic Signatures of Cultured IPF Patient Cells Reflect Gene Expression Changes Seen in Single-Cell RNA Sequencing Data

To confirm that the cells used in this experiment reflected the transcriptomic characteristics of their native tissue, the list of differentially expressed genes for the IPF patient-derived cell cultures (relative to the non-IPF controls) was compared to single-cell RNA sequencing data. The list of significant DEGs from the IPF vs. non-IPF control comparison showed increased fold changes for *ABCC5-AS1*, *DYNAP*, and *HMMR* (Supplementary Figure S1). These patterns align with the information gathered in the IPF Cell Atlas, indicating upregulation of all these genes in ciliated cells from individuals with IPF.

#### 3.2.6. RT-PCR Shows an Increase in FOXM1 in the IPF Cell Cultures

To confirm the results of the differential gene expression analysis, RT-PCR was performed on non-IPF control and IPF cell cultures for the fibrosis marker *FOXM1*. *FOXM1* is expressed more highly in fibrotic tissues and was predicted to be elevated in the upstream regulator results for the IPF common genes. An unpaired t-test comparing the two cell cultures found that the expression of *FOXM1* was significantly higher in the IPF cell cultures (Figure 3E).

### 3.3. Determination of Post-COVID Fibrosis Transcriptomic Signature

#### 3.3.1. Gene Expression Differences Are Observed between Post-COVID Fibrosis Cells and Non-IPF Control Small Airway Cells Cultured from Human Patients

**Figure 4:**
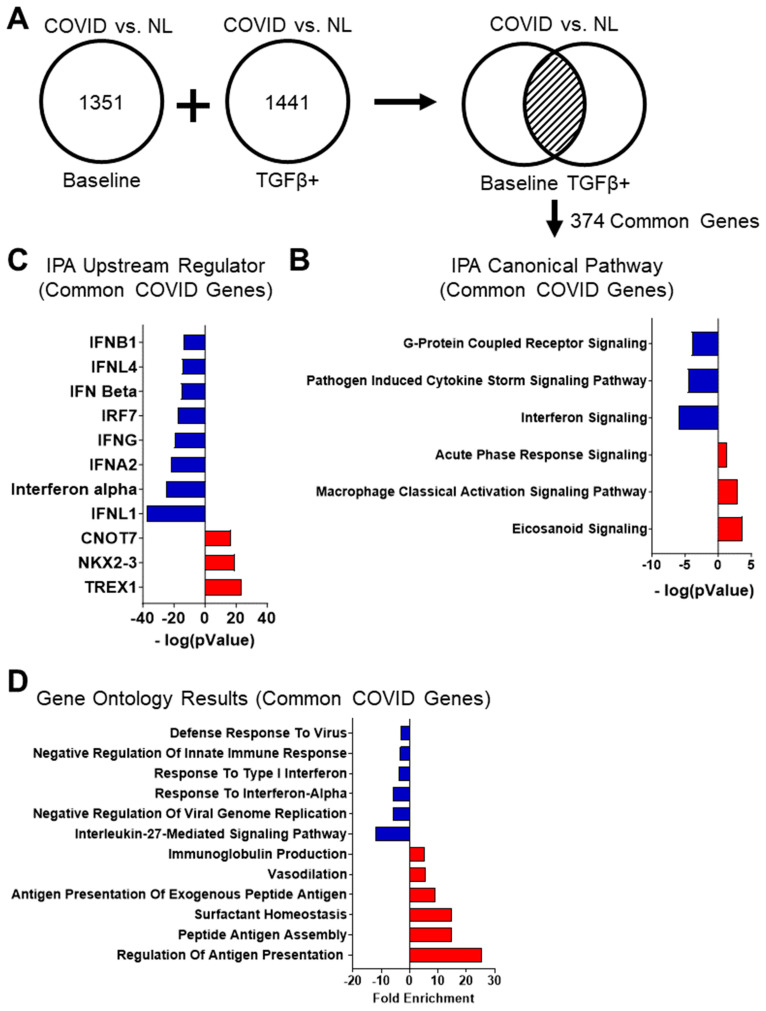
Analysis of the transcriptomic disease signature for small airway cells cultured from post-COVID fibrosis patients. (**A**) Schematic showing the determination of common DEGs for the post-COVID fibrosis vs. NL comparison. The transcriptomic signature was found by comparing the DEGs between post-COVID fibrosis lungs and non-IPF control cells in both TGF-β1 treated and untreated culture conditions. The common genes were then used for downstream analyses. (**B**) IPA canonical pathway results for the common post-COVID fibrosis cell culture genes relative to the non-IPF control samples. (**C**) IPA upstream regulator results for common post-COVID fibrosis cell culture genes relative to the non-IPF control samples. (**D**) Gene ontology results of the common genes in the post-COVID fibrosis small airway cell cultures.

The sequencing results from the post-COVID fibrosis small airway cells were compared to the data from non-IPF control small airway cell cultures. Between the baseline cultures and TGF-β1-treated culture conditions, there were 374 shared genes (Figure 4A).

#### 3.3.2. Ingenuity Pathway Analysis of Post-COVID Fibrosis vs. Non-IPF Control Small Airway Cultures Demonstrates a Trend toward Host Immune Response and Interferon Signaling

A list of 374 common DEGs between the baseline and TGF-β1-treated post-COVID fibrosis cultures was submitted for pathway analysis using the IPA tool from QIAGEN (Figure 4B and Supplementary Table S7). Each pathway was assigned an activation state and a significance value based on the fold changes of the input genes. Interferon signaling and pathogen-induced cytokine storm signaling pathways were the most significantly inhibited pathways. The activated pathways encompassed eicosanoid signaling and the macrophage classical activation signaling pathway, both of which are associated with the innate immune response.

#### 3.3.3. Upstream Regulator Results for Post-COVID Fibrosis Common Genes Show Predicted Activation of the Immune Response but Not Cytokine-Related Molecules

A list of common post-COVID fibrosis DEGs was used for upstream regulator analysis (Figure 4C and Supplementary Table S8). Upstream regulators involved in various components of the innate immune response were predicted to be activated, such as CNOT7, NKX2-3, and TREX1. Interestingly, upstream regulators related to the role of cytokines in the immune response (IFNL1, the interferon alpha family, IRF7, and IFN beta) were all predicted to be inhibited. This finding reflects the inhibition of the pathogen-induced cytokine storm signaling pathway seen in the pathway analysis of the common post-COVID fibrosis genes.

#### 3.3.4. Post-COVID Fibrosis Gene Ontology Results Show Activation of Host Immune Response and Decrease in Cilium Movement and DNA Repair

Gene ontologies for the common upregulated post-COVID fibrosis genes revealed a slight trend towards the adaptive immune system, with two entries related to antigen processing and presentation (Figure 4D and Supplementary Table S9). Also present was immunoglobulin production, another part of the body’s overall immune response. Surfactant homeostasis and vasodilation were other ontologies of note. The gene ontologies for the downregulated post-COVID fibrosis genes included several entries related to DNA repair and replication, such as double-strand break repair via break-induced replication, DNA replication initiation, and the regulation of DNA-templated DNA replication (Supplementary Table S10). Downregulated gene ontologies demonstrated a predicted inhibitory effect of cilium movement with outer dynein arm assembly, epithelial cilium movement involved in extracellular fluid movement, sperm flagellum assembly, and regulation of the microtubule-based process. Entries related to the immune system are in the downregulated gene ontologies, including responses to various interferons.

### 3.4. Identification of Crucial Differences between IPF and Post-COVID Fibrosis Small Airway Cell Cultures

#### 3.4.1. Differential Gene Expression Analysis of IPF vs. Post-COVID Fibrosis Shows Common Genes between Baseline Cultures and TGF-β1 Culture Conditions

**Figure 5:**
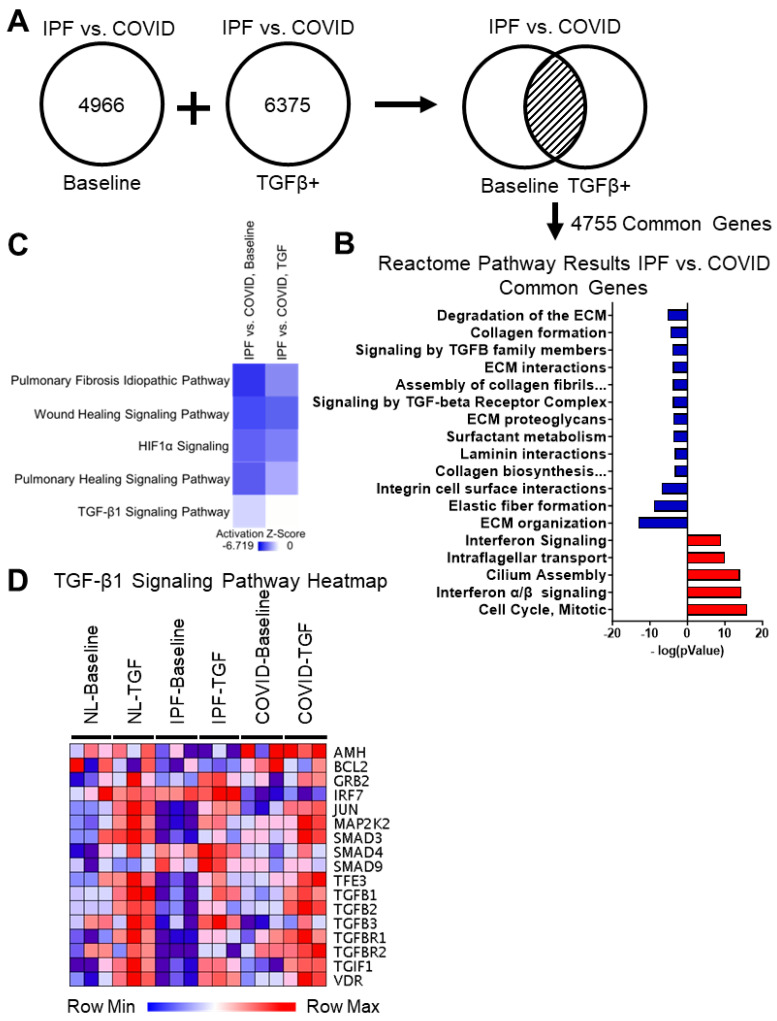
The comparison of IPF and post-COVID fibrosis gene signatures. (**A**) Schematic showing the determination of common DEGs from the IPF vs. post-COVID fibrosis comparison. The transcriptomic signature was found by comparing the DEGs between IPF lungs and post-COVID fibrosis samples in both TGF-β1 treated and untreated culture conditions. The common genes were then used for downstream analyses. (**B**) Reactome pathway analysis results for IPF vs. COVID common genes; results reflect pathway activation/inhibition in the IPF cell cultures. (**C**) IPA canonical pathway results comparing the DEG lists for the IPF vs. COVID comparison under control and TGF-β1 treatment conditions; results reflect the inhibition of pathways observed in the IPF cell cultures compared to the post-COVID fibrosis samples. (**D**) Heatmap displaying gene expression levels for genes involved in the TGF-β1 signaling pathway, a key driver of fibrosis. (NL = “normal lung”/non-IPF control, IPF = idiopathic pulmonary fibrosis, COVID = post-COVID fibrosis).

Differential gene expression analysis was performed on the IPF vs. post-COVID fibrosis RNA-sequencing data. Under baseline conditions, 4966 DEGs were shared between the IPF and post-COVID fibrosis samples; under the TGF-β1 treated samples, there were 6375 genes (Figure 5A). Of particular interest were the 4755 genes that were differentially expressed under both culture conditions. These genes have the potential to play essential roles in the development of the diseases’ phenotypes.

#### 3.4.2. Reactome Pathway Results for the IPF vs. Post-COVID Fibrosis Common Genes Show Upregulation in Interferon Signaling, Microtubule Organization, and Cell Cycle in the IPF Cell Cultures Compared to the Post-COVID Fibrosis

The 4755 DEGs shared by both culture conditions for the IPF vs. post-COVID fibrosis comparison were filtered by significance (*p*-value ≤ 0.01) and separated by fold change. Significant DEGs with a positive fold change were submitted to Reactome.org for pathway analysis (Figure 5B and Supplementary Table S11). A study of the upregulated genes showed pathways related to interferon signaling, intraflagellar transport, cilium assembly, and the mitotic cell cycle.

#### 3.4.3. Reactome Pathway Results for the IPF vs. Post-COVID Fibrosis Common Genes Show Downregulation in Extracellular Matrix Assembly, Cell-Surface Interactions, and Integrin Signaling in the IPF Cell Cultures

Like the method described previously, significant DEGs with a negative fold change resulting from the IPF vs. post-COVID fibrosis comparison were used as input for pathway analysis on Reactome.org (Figure 5B and Supplementary Table S12). Pathways associated with the downregulated genes in the IPF cell cultures included extracellular matrix-associated pathways, such as collagen biosynthesis and modifying enzymes, ECM proteoglycans, laminin interactions, assembly of collagen fibrils, collagen formation, and others.

#### 3.4.4. Comparing Gene Expression in Small Airway Cell Cultures from IPF and Post-COVID Fibrosis Reveals Distinctions in Baseline TGF-β1 Signaling

The treatment conditions for the IPF vs. COVID cultures were compared using the pathway analysis tool from IPA. The list of DEGs for each condition was used as input, and an activation z-score was calculated to compare the effect of the TGF-β1 treatment on the small airway cell cultures (Figure 5C and Supplementary Table S13). The comparison showed a lower activation z-score for pathways associated with fibrosis, such as the pulmonary fibrosis idiopathic and the pulmonary healing signaling pathways, in the untreated baseline cultures. The TGF-β1 signaling pathway also had a lower z-score in the baseline/untreated control cultures.

Genes from the TGF-β1signaling pathway were selected for gene expression analysis (Figure 5D). The read count datasets from IPF and post-COVID fibrosis samples were visualized using a heatmap based on TGF-β1 signaling pathway genes. In non-IPF control lung samples, most genes associated with the TGF-β1 signaling pathway were expressed at low levels under the baseline condition; examples of these molecules include *JUN*, *MAP2K2*, *TGF-β1*, and *TGF-β2*. For the IPF lung samples, the baseline expression levels of many of the genes were low, even compared to the non-IPF lung samples. The baseline expression of TGF-β1 signaling pathway-related genes was moderately decreased in the untreated post-COVID fibrosis samples, though levels were still higher than the IPF sample baseline. Treating the post-COVID fibrosis cultures with TGF-β1 increased the expression of selected genes.

#### 3.4.5. Differences in BMP Signaling Are Observed between Post-COVID Fibrosis and IPF Cell Cultures

**Figure 6:**
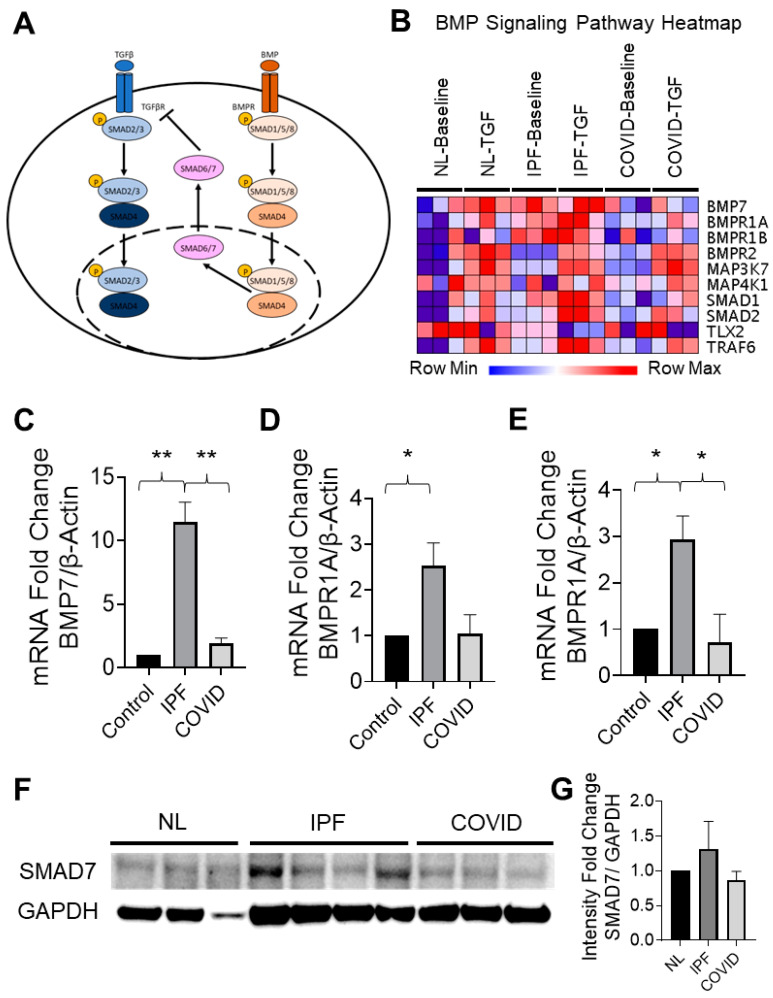
Evaluation of BMP signaling in patient-derived small airway cell cultures. (**A**) Mechanism of inhibition of TGFβ signaling by BMP. (**B**) Heatmap displaying the expression levels of genes involved in the BMP signaling pathway (NL = “normal lung”/non-IPF control, IPF = idiopathic pulmonary fibrosis, COVID = post-COVID fibrosis). (**C**–**E**) RT-PCR quantification of fold changes for genes associated with the BMP signaling pathway in small airway cell cultures (** indicates *p*-value < 0.001, * indicates *p*-value < 0.05, *N* = 3). (**F**) Western blot showing the detection of the SMAD7 protein in human normal (NL), IPF, and post-COVID fibrosis (COVID) lung tissue. (**G**) Quantification of SMAD7 protein levels in normal lung (*N* = 3), IPF (*N* = 4), and post-COVID fibrosis (*N* = 3) lung tissue.

The possible effect of BMP signaling on TGF-β1 signaling in the cell cultures was explored. The BMP signaling pathway is a known inhibitor of the TGF-β1 signaling pathway (Figure 6A) [26]. Expression levels of genes involved in the BMP signaling pathway were visualized across the samples with a heatmap (Figure 6B). The analysis of these genes showed higher expression in the IPF samples than in the post-COVID fibrosis samples; *BMP7*, *BMPR1A*, *BMPR1B*, *SMAD1*, and *SMAD2* all demonstrated this expression pattern. RT-PCR was used to validate this observation in small airway cell culture samples (Figure 6C–E). The fold changes for *BMP7* and *BMPR1B* were significantly higher in the IPF samples when compared to the non-IPF lung and post-COVID fibrosis samples. To further support the hypothesis that BMP signaling inhibited the fibrotic activity of TGF-β1 signaling, Western blotting was used to detect total levels of the SMAD7 protein in normal, IPF, and post-COVID fibrosis distal lung tissue (Figure 6F). Quantification of the Western blot revealed an increase in SMAD7 protein in the IPF samples that trended towards significance (Figure 6G).

#### 3.4.6. pSMAD1/5/8 Expression and Nuclear Localization in Normal, IPF, and Post-COVID Fibrosis Tissues

**Figure 7:**
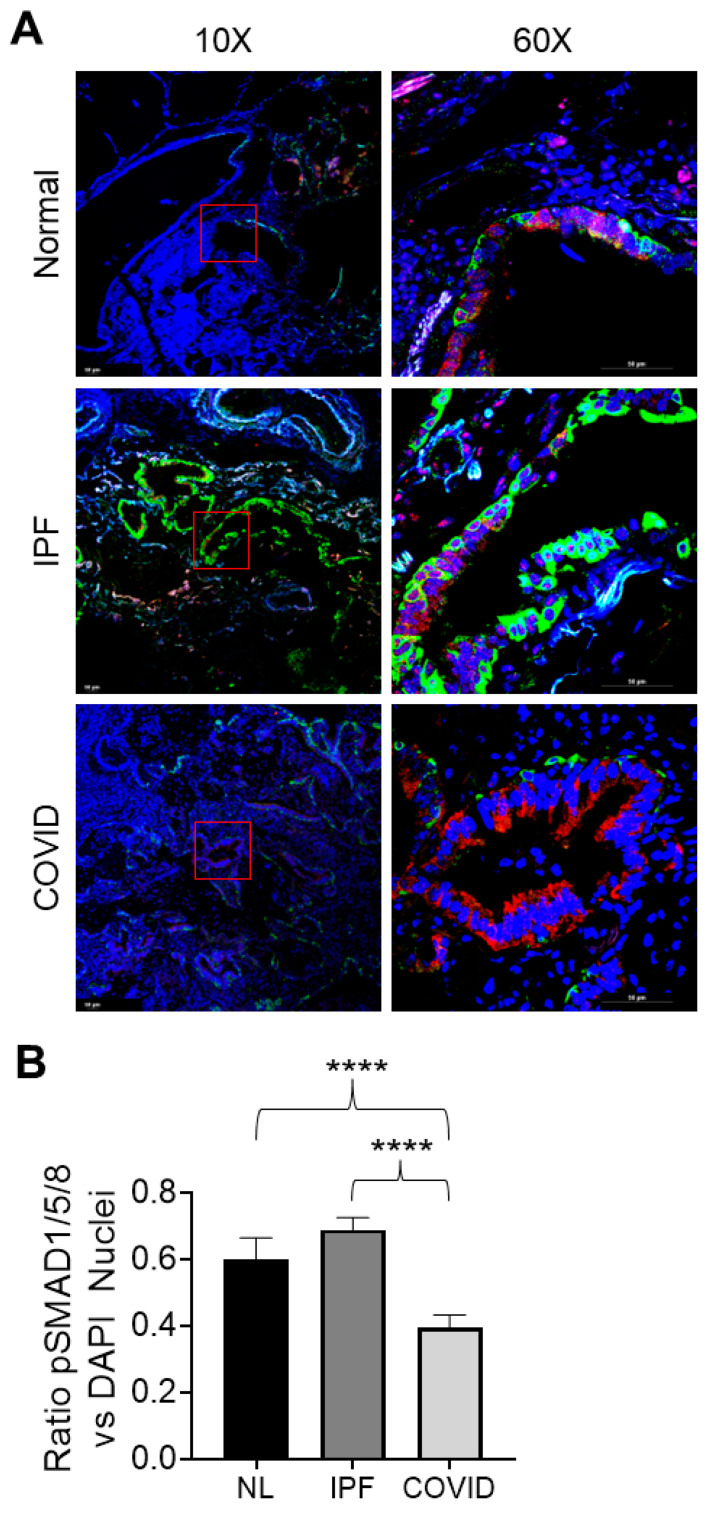
Localization of pSMAD1/5/8 to the nucleus in the patient sample tissue. (**A**) IHC images of patient samples (IPF = idiopathic pulmonary fibrosis, COVID = post-COVID fibrosis) stained for DAPI (blue), pSMAD1/5/8 (red), and the epithelial cell marker KRT5 (green). The scale bar was equal to 50 μM. Images are shown at two different magnifications; the red square in the 10× images indicates the region of interest pictured in the 60× image. (**B**) Quantification of the nuclear localization of pSMAD1/5/8 across different tissue types (**** indicates *p*-value < 0.0001).

The expression of *pSMAD1/5/8*, a regulator of BMP signaling, was visualized in the normal, IPF, and post-COVID fibrosis tissues using immunofluorescence (Figure 7A). Amongst the three tissue types, the IPF samples demonstrated higher levels of pSMAD1/5/8 nuclear localization (Figure 6B). The levels of nuclear localization were the lowest in the post-COVID fibrosis tissue, followed by normal lung tissue. Calculation of the *p*-values showed that the nuclear localization of pSMAD1/5/8 was significantly higher in the IPF samples compared to the post-COVID fibrosis and normal lung samples.

## 4. Discussion

Idiopathic pulmonary fibrosis (IPF) is a devastating disease that impairs gas exchange in the lungs and inhibits normal lung function. It is the most common idiopathic interstitial pneumonia (IIP), accounting for 20–50% of chronic interstitial lung disease cases [27]. Some of the hallmarks of IPF include the destruction of the air sacs in the lung (alveoli), the accumulation of scar tissue, and abnormal morphology changes in the airways [3]. TGF-β1 has been shown to play an essential role in the pathogenesis of IPF, influencing disease characteristics such as myofibroblast accumulation and collagen deposition [14]. Research suggests that specific environmental factors, cigarette smoking, and infections may elevate the risk of acquiring this disease [27].

Pulmonary fibrosis is a significant risk factor in developing complications following COVID-19 infection [12]. Patients with interstitial lung diseases, such as pulmonary fibrosis, are at a higher risk of death following infection with COVID-19 than patients without underlying respiratory diseases [12]. Acute lung injury, such as infection, can exacerbate pre-existing IPF [13]. Pathological characteristics shared between COVID-19 and pulmonary fibrosis patients include the accumulation of fibrin in the alveolar spaces, the deterioration of vascular systems, and the buildup of collagen and other extracellular matrix components [9]. This study used bulk RNA-sequencing techniques to build IPF and post-COVID fibrosis transcriptomic disease signatures.

### 4.1. Patient-Derived Small Airway Cell Cultures Maintain Tissue-Specific Markers and Reflect Gene Expression Patterns Observed in Fibrotic Lung Tissue

This research used small airway cultures from lung tissues affected by IPF and post-COVID fibrosis. The cells grown on the transwell membranes showed evidence of ciliated cells (Figure 2A). Notably, the cultures from non-IPF lungs had the highest percentage of ciliated cells. The study found that SFTPA1, linked to early mortality in IPF patients, was expressed only in small airway cultures, indicating that they maintain characteristics of the original tissue [3]. Few studies focus on small airways in IPF. Comparing gene expression in cultured cells with an IPF dataset revealed similarities, suggesting that these cells mirror traits of the native fibrotic tissue. Ciliated cell markers and fibrosis-related gene expression further support this.

Very few studies have investigated the role of small airways in idiopathic pulmonary fibrosis (IPF). To ensure that the cultured cells reflected the characteristics of the original fibrotic lung tissue, the genes expressed in IPF compared to normal lungs were examined. The analysis was performed by comparing differentially expressed genes (DEGs) in the IPF vs. NL dataset with the IPF Cell Atlas, an online resource of RNA data for various cell types related to IPF (Supplementary Figure S7) [28]. The comparison revealed upregulated genes in untreated IPF samples, specifically in ciliated cells, as seen in the IPF Cell Atlas. Similarities between the Cell Atlas and gene expression in the IPF vs. NL dataset suggest that the patient-derived cells exhibit traits of their native fibrotic tissue. Markers for ciliated cells and the expression of fibrosis-related genes implied that the harvested cells retain characteristics of the original tissue.

### 4.2. Transcriptomic Signatures of IPF Cell Cultures Include the Activation of Cell Cycle Regulators, the Upregulation of Ciliary Movement, and the Predicted Inhibition of the Pulmonary Fibrosis Idiopathic and TGF-β1 Signaling Pathways

A total of 1991 differentially expressed genes (DEGs) were found in both the untreated and TGFβ-treated conditions of small airway cell cultures in IPF (Figure 3A). The kinetochore metaphase signaling pathway was the most significant, suggesting activation in IPF cultures and the expected activation of cyclins and cell cycle regulation (Figure 3B). Evidence, including the upregulation of cell cycle regulators ELDR, CKAP2L, PCLAF, and the significant increase in *FOXM1* expression, pointed to a potential role of cell cycle control in IPF development (Figure 3C,E). FOXM1, a transcription factor linked to pulmonary diseases, may influence cell cycle transitions and has associations with lung injury repair and the development of pulmonary fibrosis [29]. The presence of ontologies related to the cell cycle and the upregulation of *FOXM1* highlighted the crucial role of cell cycle regulation in IPF development.

Also present in the IPF datasets were various ontologies related to ciliary movement (Figure 3D). Cilia are hair-like projections in the inner lining of airways that help move water, mucus, and debris. A study published in 2018 found that IPF patient tissue demonstrated an increased number of primary cilia compared to normal lung tissue [30]. The bulk RNA-sequencing data collected by this study seemed to reflect this increase in cilia with an increase in ontologies related to cilia movement and transportation.

The gene activity observed in IPF cell cultures indicated the suppression of fibrosis-associated pathways. More precisely, comparisons between IPF and normal datasets predicted the inhibition of signaling pathways related to idiopathic pulmonary fibrosis and wound healing. The TGF-β family, including TGF-β1, exhibited negative activation scores calculated by IPA software (QIAGEN Inc., (Version 101138820) https://digitalinsights.qiagen.com/IPA, accessed on 18 October 2023), and we delve into this further in the upcoming sections.

### 4.3. The Disease Signature of Post-COVID Fibrosis Illustrates a Distinct Interplay of Activation and Inhibition within Components of the Immune System

The comparison of post-COVID fibrosis patient cell cultures found 374 shared genes between baseline and TGF-β1 treated cultures (Figure 4A). The IPA software predicted the activation of the macrophage activation pathway, which is crucial for the immune system (Figure 4B). Gene ontology results indicated active macrophage-mediated signaling in epithelial cells, with entries related to antigen processing and presentation (Figure 4D). The upregulation of major histocompatibility complex (MHC) proteins, vital for the adaptive immune system, was also observed among the upregulated gene ontologies (Supplementary Table S9) [31].

The acute phase response signaling pathway was predicted to be activated among the canonical pathways. This pathway represents the body’s system-wide reaction to a physiological threat, such as injury or infection. The acute phase response triggers the synthesis of acute phase reaction proteins (APRPs) that can be used as markers of inflammation [32,33]. Several studies have found that serum levels of APRPs can be used as diagnostic indicators of COVID-19 infection severity when taken in conjunction with patient symptoms [32,33]. The predicted activation of immune system components in the COVID vs. NL comparison reflected these previous studies.

Contrary to the expected immune system activation, there was the predicted inhibition of interferon signaling and the pathway for cytokine storms induced by pathogens in post-COVID fibrosis cell cultures. Interferons, part of the cytokine family, are linked to immune responses. Still, their connection with lipid-based eicosanoids (a prominently predicted pathway) is not fully understood [34]. Like macrophages, cytokines are crucial for immune responses. The predicted inhibition of regulators like IRF7 and IFNL1 supports interferon and cytokine signaling inhibition (Figure 4C). IRF7 aids the immune system in responding to viral threats, while IFNL1 belongs to the cytokine family [35,36]. Gene ontology results for common post-COVID fibrosis genes showed upregulation in antigen presentation and immunoglobulin production but downregulation in response to Type I interferon, defense against viruses, and response to interferon-alpha (Figure 4D). The cellular mechanisms behind these findings are left for future investigations.

### 4.4. Comparison of IPF vs. Post-COVID Fibrosis DEGs Highlights Mucociliary Dysfunction in Post-COVID Fibrosis Cell Cultures Compared to IPF

This study aimed to uncover differences in gene activity between small airway cell cultures of idiopathic pulmonary fibrosis (IPF) and post-COVID fibrosis. Analyzing 4755 genes differentially expressed in both untreated and TGF-β1 treated conditions, which may have future clinical importance, was a focus. The Reactome pathway analysis revealed increased cilium assembly and intra-flagellar transport in IPF cultures compared to post-COVID fibrosis cultures (Figure 5B). Ciliated cells are crucial for respiratory defense, preventing mucus buildup. A 2021 study noted that COVID-19 infection leads to ciliary cell loss and impaired mucociliary clearance, with the virus replicating in airway epithelia [37].

### 4.5. IPF Cell Cultures Demonstrate a Decrease in Extracellular Matrix-Associated Pathways and Collagen Fiber Biosynthesis Compared to Post-COVID Fibrosis Cell Cultures

COVID-19 infection in airway tissue can cause an abnormal buildup of extracellular matrix (ECM) components [38]. By examining IPF versus post-COVID fibrosis data, the Reactome pathway analysis revealed decreased ECM-related pathways in IPF cell cultures (Figure 5B). Proteoglycans, vital for inflammation and fibrosis development, play a role in ECM [38]. Unregulated wound healing leads to collagen accumulation, fibrosis, and lung scarring. Pathway results suggest ECM dysregulation in post-COVID fibrosis, indicating increased elastic fiber and collagen formation, cell-ECM interactions, and collagen biosynthesis.

### 4.6. IPF Small Airway Cell Cultures Demonstrate Lower Levels of Baseline TGF-β1 Signaling

The Reactome pathway analysis showed reduced genes related to TGFβ family signaling in IPF cell cultures compared to post-COVID fibrosis (Figure 5B). TGF-β1, a critical factor in fibrosis, is released in response to threats or injury. Elevated TGF-β1 levels are found in pulmonary fibrosis and acute-phase COVID-19 [16]. The predicted inhibition of fibrosis-related pathways, including TGF-β1 signaling, in IPF cultures prompted a closer look (Figure 5C). A comparison with COVID samples revealed predicted inhibition in various fibrosis-related pathways (Figure 5C). Interestingly, TGF-β1-treated IPF cultures had higher activation scores than baseline, prompting a gene expression analysis. The study found that crucial genes in the TGF-β1 signaling pathway were expressed at lower levels in IPF cultures, particularly in untreated samples, compared to post-COVID fibrosis (Figure 5D).

### 4.7. Higher Levels of BMP Signaling in IPF Samples Decrease TGF-β1 Signaling and Contribute to the Predicted Inhibition of Key Fibrosis-Related Pathways

The TGF-β1 and BMP signaling pathways have a delicate balance, with interactions that can either hinder or promote each other [39]. SMAD4 is crucial for this equilibrium, facilitating the translocation of BMP-related SMAD1/5/8 into the nucleus, inhibiting TGF-β1 signaling (Figure 6A) [39]. Studies show that BMPs can protect lungs from TGF-β1-induced fibrosis [39]. IPF samples exhibit higher BMP signaling genes, confirmed by RT-PCR (Figure 6C–E). The proposed TGF-β1 inhibition mechanism involves BMP-induced SMAD7 activation. SMAD7 inhibits TGF-β1 signaling and fibrotic responses [39]. IPF patients’ lungs showed high SMAD7 levels [40]. Post-COVID fibrosis samples had lower BMP signaling, potentially explaining their need for earlier transplants than non-COVID-19 IPF patients.

Both normal and IPF samples showed increased nuclear localization of pSMAD1/5/8, confirmed by quantifying SMAD1/5/8-positive nuclei (Figure 7A,B). In contrast, post-COVID fibrosis samples had pSMAD1/5/8 in the cytoplasm. The translocation of the pSMAD1/5/8-SMAD4 complex to the nucleus released SMAD6/7 into the cytoplasm, inhibiting TGF-β1 receptors. Elevated pSMAD1/5/8 in IPF nuclei may indicate higher BMP signaling, potentially a feedback response to increased TGF-β1 levels. Lower BMP signaling in post-COVID lung may explain the patients’ need for earlier transplants than the non-COVID-19 IPF patients. Further studies are required to explore this mechanism in detail.

## 5. Study Limitations

In this study, the authors acknowledge some limitations. We used lung tissue from patients undergoing lobectomies who did not have IPF before their surgery, some of whom had a smoking history. The RNA-sequencing samples came from non-IPF control subjects with a smoking history, making them relevant for comparison. However, the study had a limited number of samples and patients. The authors harvested cells from small airway tissue and cultured them for at least three weeks. We recognize that the corticosteroids included in the medium could affect the cellular gene expression, but care was taken to ensure that all cultures were treated identically. The study treated cultures with TGF-β1 to mimic in vivo conditions for 48 h. The authors compared TGF-β1-treated samples to minimize any biased effects. The transcriptomic trends seen in TGF-β1-treated cultures were also present in baseline conditions. Culturing small airways is challenging, but cells derived from this method maintain the characteristics of the native tissue. Despite potential issues with culturing, the data showed clear transcriptomic patterns, as bulk RNA-sequencing covers a more complete transcriptomic profile than single-cell RNA-sequencing. Further research is needed to understand the differences in gene expression between IPF and post-COVID fibrosis cell cultures.

## 6. Conclusions

This research looked at the transcriptomic signatures of cultured small airways derived from IPF and post-COVID fibrosis. These cells mimic the characteristics of fibrotic tissues, making them a suitable model. The study found that post-COVID fibrosis cells had a different transcriptomic signature than IPF cells. In post-COVID fibrosis, genes related to the restructuring of the ECM and tissue repair were more highly expressed than in the IPF cell cultures. Additionally, these cells were more responsive to treatment with TGF-β1. Cell cultures and tissue from IPF patients demonstrated elevated BMP signaling levels when compared to post-COVID fibrosis. Understanding these distinctions may lead to improved therapies for both diseases.

## Data Availability

All data presented in this study are available upon request from the corresponding authors. The data collected from RNA sequencing have been made available in the Gene Expression Omnibus database (GSE225549).

## Supplementary Materials

The following supporting information can be downloaded at: https://www.mdpi.com/article/10.3390/cells12202501/s1, Figure S1: scRNA sequencing results from the IPF Cell Atlas; Figure S7: Comparison of IPF and post-COVID fibrosis gene signatures under baseline conditions; Table S1: Clinical data summary; Table S2: List of primer sequences used for RT-PCR analysis; Table S3: Top 100 canonical pathway analysis results for the IPF vs. Normal DEG comparison; Table S4: Top 100 upstream regulator results for the IPF vs. Normal DEG comparison; Table S5: Top 100 gene ontology results for the IPF vs. Normal upregulated DEGs; Table S6: Top 100 gene ontology results for the downregulated DEGs in for the IPF vs. Normal comparison; Table S7: Top 100 canonical pathway analysis results for the COVID vs. Normal DEG comparison; Table S8: Top 100 upstream regulator results for the COVID vs. Normal DEG comparison; Table S9: Top 100 gene ontology results for the upregulated DEGs in for the COVID vs. Normal comparison; Table S10: Top 100 gene ontology results for the downregulated DEGs in for the COVID vs. Normal comparison; Table S11: Reactome pathway results for the upregulated genes in the IPF vs. COVID dataset; Table S12: Reactome pathway results for the downregulated genes in the IPF vs. COVID dataset; Table S13: IPA canonical pathway comparison between the baseline and TGF-β-treated cultures in the IPF vs. COVID dataset; Table S14: Glossary of commonly used terms and abbreviations found in the manuscript.

## Abbreviations

ILD	Interstitial lung disease
IPF	Idiopathic pulmonary fibrosis
COPD	Chronic obstructive pulmonary disease
SARS-CoV-2, COVID-19	Severe acute respiratory syndrome coronavirus 2
TGF-β1	Transforming growth factor beta
