# Supplementary material for: Differential Transcriptomic Signatures of Small Airway Cell Cultures Derived from IPF and COVID-19-Induced Exacerbation of Interstitial Lung Disease
Source: Cells. 2023 Oct 21;12(20):2501. doi: 10.3390/cells12202501 (PMC10605205; doi:10.3390/cells12202501)
Supplement: Supplementary file 1 [file cells-12-02501-s001.zip › cells-2614249-supplementary/Figure S1.pdf]

A

Gene Name	log_2Fold Change	pValue	Adjusted pValue
ABCC5-AS1	8.3793	1.75E-07	0.000615
DYNAP	8.7718	1.95E-07	0.000615
HMMR	4.642	8.40E-06	0.009467

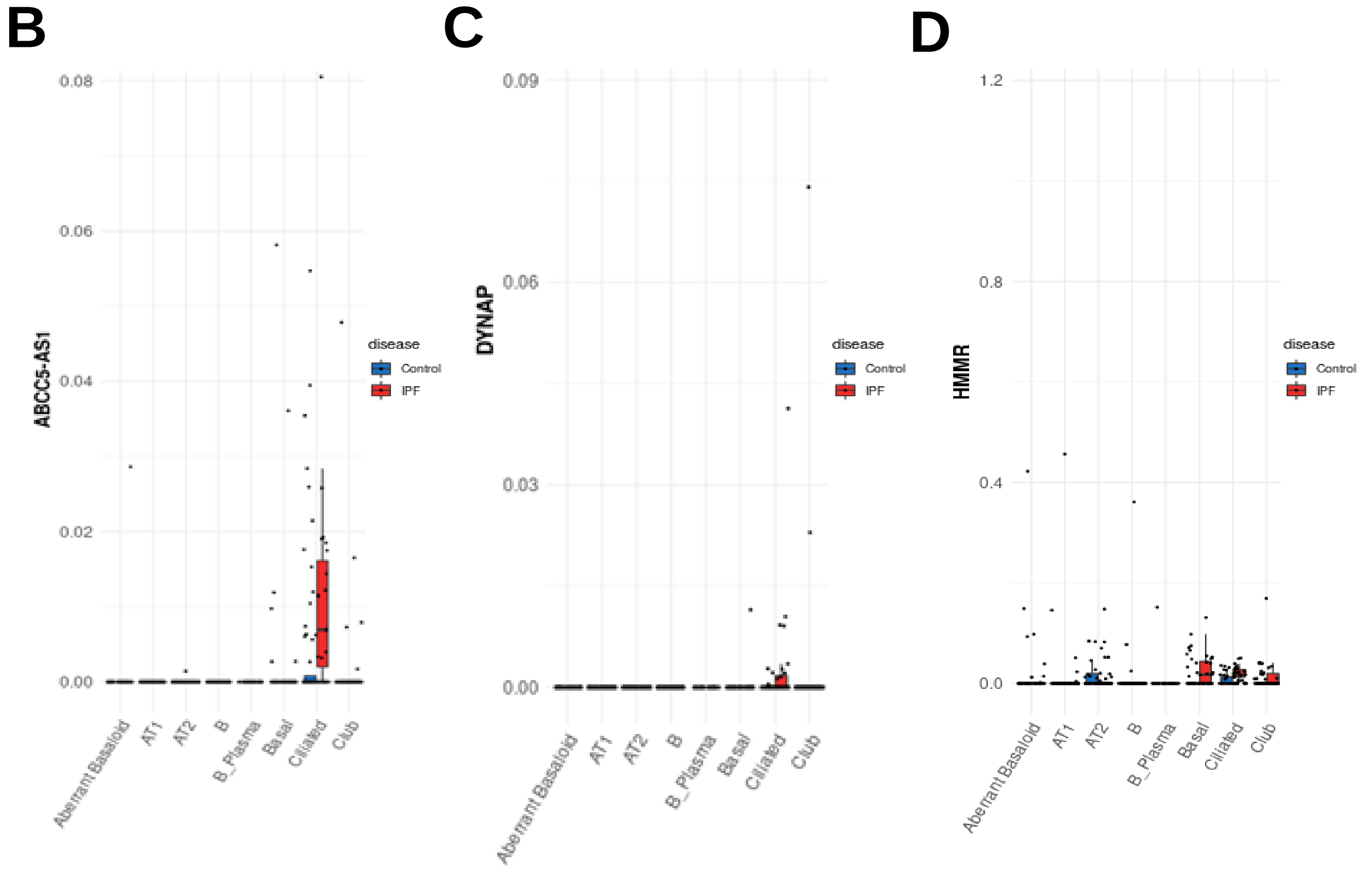

Supplementary Figure 7. scRNA sequencing results from the IPF Cell Atlas. (A) DEG results for the IPF vs. non-IPF control baseling comparison. **(B)** IPF tissue gene expression levels for *ACC5-AS1* organized by cell type and disease state. **(C)** IPF tissue gene expression levels for *DYNAP* organized by cell type and disease state. **(D)** IPF tissue gene expression levels for *HMMR* organized by cell type and disease state. Note: The graphs shown have been adapted from the data representations generated by the IPF Cell Atlas for the Kaminski/Rosas dataset (http://www.ipfcellatlas.com/).
