# Supplementary material for: Differential Transcriptomic Signatures of Small Airway Cell Cultures Derived from IPF and COVID-19-Induced Exacerbation of Interstitial Lung Disease
Source: Cells. 2023 Oct 21;12(20):2501. doi: 10.3390/cells12202501 (PMC10605205; doi:10.3390/cells12202501)
Supplement: Supplementary file 1 [file cells-12-02501-s001.zip › cells-2614249-supplementary/Figure S2.pdf]

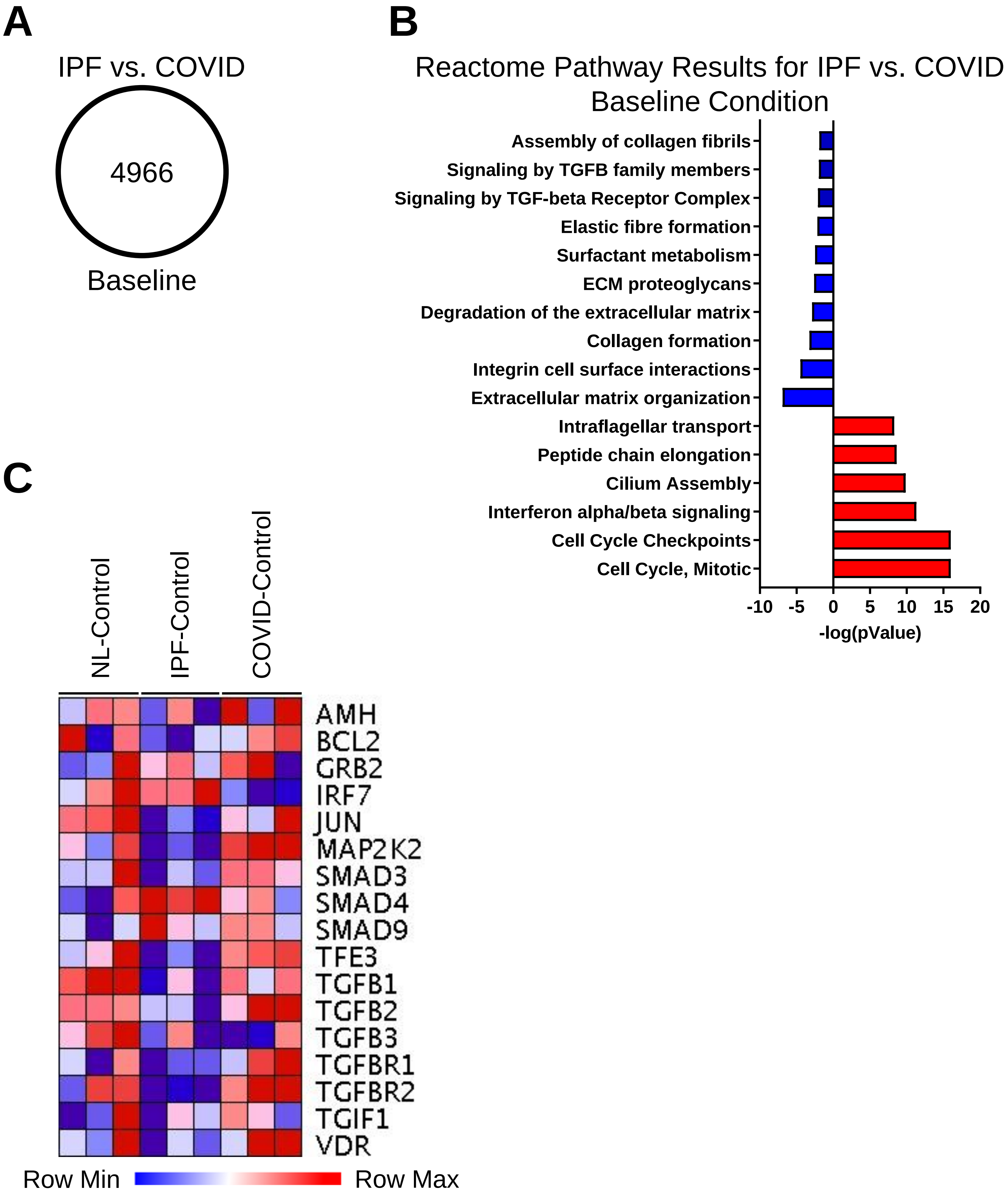

Supplementary Figure 15. Comparison of IPF and post-COVID fibrosis gene signatures under baseline conditions. (A) A total of 4,966 DEGs were identified when comparing the baseline (untreated) IPF cell cultures to the post-COVID fibrosis cell cultures. **(B)** Reactome pathway analysis results for the IPF vs. COVID comparison under baseline conditions. **(C)** Heatmap displaying gene expression levels for genes involved in the TGF-β1 signaling pathway, a key driver of fibrosis. (NL= “normal lung”/non-IPF control, IPF = idiopathic pulmonary fibrosis, COVID = post-COVID fibrosis).
