# Supplementary material for: Differential Transcriptomic Signatures of Small Airway Cell Cultures Derived from IPF and COVID-19-Induced Exacerbation of Interstitial Lung Disease
Source: Cells. 2023 Oct 21;12(20):2501. doi: 10.3390/cells12202501 (PMC10605205; doi:10.3390/cells12202501)
Supplement: Supplementary file 1 [file cells-12-02501-s001.zip › cells-2614249-supplementary/Table S10.pdf]

Supplementary Table S10. Top 100 gene ontology results for the downregulated DEGs in for the COVID vs. Normal comparison.

GO biological process complete	Fold Enrichment	Raw P-value	FDR
rhombomere development (GO:0021546)	11.95	3.22E-04	4.03E-02
interleukin-27-mediated signaling pathway (GO:0070106)	11.95	3.22E-04	4.00E-02
double-strand break repair via break-induced replication (GO:0000727)	9.76	4.94E-05	8.71E-03
mitotic DNA replication (GO:1902969)	8.36	3.28E-04	4.06E-02
DNA unwinding involved in DNA replication (GO:0006268)	6.84	3.66E-05	6.92E-03
outer dynein arm assembly (GO:0036158)	6.54	4.84E-05	8.62E-03
DNA replication initiation (GO:0006270)	6.34	8.94E-06	2.42E-03
nuclear DNA replication (GO:0033260)	6.27	6.32E-05	1.07E-02
cell cycle DNA replication (GO:0044786)	6.02	8.17E-05	1.28E-02
negative regulation of viral genome replication (GO:0045071)	5.87	6.17E-09	6.92E-06
axonemal dynein complex assembly (GO:0070286)	5.85	1.19E-06	4.66E-04
response to interferon-alpha (GO:0035455)	5.82	2.47E-04	3.20E-02
motile cilium assembly (GO:0044458)	5.4	1.99E-08	1.73E-05
axoneme assembly (GO:0035082)	5.28	9.42E-12	1.85E-08
epithelial cilium movement involved in extracellular fluid movement (GO:0003351)	4.78	3.72E-05	6.94E-03
sperm flagellum assembly (GO:0120316)	4.65	1.99E-04	2.65E-02
extracellular transport (GO:0006858)	4.46	6.55E-05	1.09E-02

negative regulation of viral process (GO:0048525)	4.4	1.14E-08	1.19E-05
microtubule bundle formation (GO:0001578)	4.11	8.32E-10	1.00E-06
regulation of viral genome replication (GO:0045069)	4.04	5.63E-07	2.38E-04
response to type I interferon (GO:0034340)	3.86	2.11E-04	2.78E-02
regulation of DNA-templated DNA replication (GO:0090329)	3.81	1.29E-04	1.84E-02
cilium movement (GO:0003341)	3.74	1.18E-10	1.54E-07
response to vitamin (GO:0033273)	3.35	8.82E-05	1.36E-02
negative regulation of innate immune response (GO:0045824)	3.3	1.65E-04	2.27E-02
defense response to virus (GO:0051607)	3.17	6.62E-11	1.04E-07
defense response to symbiont (GO:0140546)	3.16	7.46E-11	1.06E-07
recombinational repair (GO:0000725)	3.14	1.07E-05	2.72E-03
double-strand break repair via homologous recombination (GO:0000724)	3.11	1.97E-05	4.12E-03
DNA duplex unwinding (GO:0032508)	3.09	1.26E-04	1.83E-02
DNA geometric change (GO:0032392)	3.07	8.62E-05	1.34E-02
mitotic sister chromatid segregation (GO:0000070)	3.07	1.52E-05	3.45E-03
cilium assembly (GO:0060271)	3.01	4.37E-12	1.37E-08
DNA-templated DNA replication (GO:0006261)	3.01	5.51E-06	1.60E-03
microtubule-based movement (GO:0007018)	2.95	1.37E-13	7.14E-10
cilium-dependent cell motility (GO:0060285)	2.95	1.11E-05	2.77E-03
cilium or flagellum-dependent cell motility (GO:0001539)	2.95	1.11E-05	2.73E-03
regulation of viral life cycle (GO:1903900)	2.94	7.64E-06	2.10E-03

cilium organization (GO:0044782)	2.87	6.98E-12	1.82E-08
DNA conformation change (GO:0071103)	2.81	2.28E-04	2.98E-02
regulation of viral process (GO:0050792)	2.75	9.98E-06	2.61E-03
cilium movement involved in cell motility (GO:0060294)	2.72	1.57E-04	2.18E-02
regulation of DNA replication (GO:0006275)	2.71	8.08E-05	1.28E-02
DNA replication (GO:0006260)	2.61	5.21E-06	1.54E-03
sister chromatid segregation (GO:0000819)	2.6	1.43E-04	2.02E-02
double-strand break repair (GO:0006302)	2.55	9.97E-06	2.65E-03
female gamete generation (GO:0007292)	2.53	1.74E-04	2.34E-02
microtubule cytoskeleton organization (GO:0000226)	2.45	7.13E-12	1.60E-08
response to virus (GO:0009615)	2.44	4.94E-08	3.69E-05
nuclear chromosome segregation (GO:0098813)	2.43	1.13E-05	2.72E-03
spermatid differentiation (GO:0048515)	2.37	1.06E-04	1.57E-02
spermatid development (GO:0007286)	2.36	1.48E-04	2.07E-02
plasma membrane bounded cell projection assembly (GO:0120031)	2.36	1.54E-08	1.42E-05
cell projection assembly (GO:0030031)	2.33	1.50E-08	1.47E-05
nuclear division (GO:0000280)	2.33	1.02E-06	4.21E-04
DNA recombination (GO:0006310)	2.32	3.35E-05	6.40E-03
microtubule-based process (GO:0007017)	2.32	9.28E-15	7.28E-11
chromosome segregation (GO:0007059)	2.24	1.70E-05	3.75E-03
organelle fission (GO:0048285)	2.2	4.09E-06	1.28E-03

meiotic cell cycle (GO:0051321)	2.2	7.91E-05	1.27E-02
chromosome organization (GO:0051276)	2.07	2.74E-06	9.56E-04
positive regulation of cell cycle process (GO:0090068)	2.07	3.74E-04	4.54E-02
regulation of microtubule-based process (GO:0032886)	2.06	3.02E-04	3.85E-02
germ cell development (GO:0007281)	2.04	9.66E-05	1.46E-02
cellular process involved in reproduction in multicellular organism (GO:0022412)	1.95	4.15E-05	7.58E-03
organelle assembly (GO:0070925)	1.92	2.07E-08	1.71E-05
mitotic cell cycle process (GO:1903047)	1.88	1.19E-05	2.79E-03
mitotic cell cycle (GO:0000278)	1.81	1.14E-05	2.70E-03
cell cycle process (GO:0022402)	1.73	2.83E-06	9.44E-04
cell projection organization (GO:0030030)	1.73	3.18E-08	2.49E-05
cell division (GO:0051301)	1.72	2.97E-04	3.82E-02
plasma membrane bounded cell projection organization (GO:0120036)	1.71	1.44E-07	9.41E-05
regulation of cell cycle process (GO:0010564)	1.66	1.02E-04	1.52E-02
DNA metabolic process (GO:0006259)	1.62	8.93E-05	1.36E-02
cytoskeleton organization (GO:0007010)	1.58	3.55E-06	1.14E-03
gamete generation (GO:0007276)	1.57	3.92E-04	4.69E-02
cell cycle (GO:0007049)	1.57	5.06E-06	1.53E-03
multicellular organismal reproductive process (GO:0048609)	1.54	3.11E-04	3.93E-02
defense response to other organism (GO:0098542)	1.52	1.11E-04	1.62E-02

defense response (GO:0006952)	1.43	1.28E-04	1.84E-02
response to external stimulus (GO:0009605)	1.35	2.98E-05	5.77E-03
primary metabolic process (GO:0044238)	0.85	1.71E-04	2.31E-02
organic substance metabolic process (GO:0071704)	0.84	2.38E-05	4.84E-03
regulation of metabolic process (GO:0019222)	0.83	6.85E-05	1.12E-02
nitrogen compound metabolic process (GO:0006807)	0.83	5.84E-05	1.01E-02
metabolic process (GO:0008152)	0.83	2.67E-06	9.51E-04
cellular metabolic process (GO:0044237)	0.82	1.54E-05	3.46E-03
regulation of primary metabolic process (GO:0080090)	0.81	3.93E-05	7.24E-03
regulation of nitrogen compound metabolic process (GO:0051171)	0.81	5.17E-05	9.01E-03
regulation of cellular metabolic process (GO:0031323)	0.78	4.33E-06	1.33E-03
protein metabolic process (GO:0019538)	0.78	1.70E-04	2.32E-02
regulation of gene expression (GO:0010468)	0.77	1.01E-05	2.59E-03
regulation of nucleobase-containing compound metabolic process (GO:0019219)	0.77	6.22E-05	1.06E-02
macromolecule metabolic process (GO:0043170)	0.77	2.69E-07	1.32E-04
regulation of cellular biosynthetic process (GO:0031326)	0.71	3.18E-07	1.47E-04
regulation of biosynthetic process (GO:0009889)	0.71	2.04E-07	1.19E-04
regulation of macromolecule biosynthetic process (GO:0010556)	0.71	3.79E-07	1.70E-04
regulation of RNA metabolic process (GO:0051252)	0.69	2.24E-07	1.25E-04
regulation of RNA biosynthetic process (GO:2001141)	0.69	4.95E-07	2.16E-04
regulation of DNA-templated transcription (GO:0006355)	0.68	2.55E-07	1.29E-04
