# Supplementary material for: Differential Transcriptomic Signatures of Small Airway Cell Cultures Derived from IPF and COVID-19-Induced Exacerbation of Interstitial Lung Disease
Source: Cells. 2023 Oct 21;12(20):2501. doi: 10.3390/cells12202501 (PMC10605205; doi:10.3390/cells12202501)
Supplement: Supplementary file 1 [file cells-12-02501-s001.zip › cells-2614249-supplementary/Table S14.pdf]

Supplementary Table S14. Glossary of commonly used terms and abbreviations found in the manuscript.

Term	Description
AEC	Airway epithelial cell
APRP	Acute phase reaction protein
COPD	Chronic obstructive pulmonary disease
COVID-19 Exacerbated ILD	Sample group consisting of patients diagnosed with ILD before being infected by COVID-19
DEG	Differentially expressed gene
ECMO	Extracorporeal membrane oxygenation
FEV1	Forced expiratory volume
FVC	Forced vital capacity
IIP	Idiopathic interstitial pneumonia
ILD	Interstitial lung disease
IPA	Ingenuity Pathway Analysis
IPF	Idiopathic pulmonary fibrosis
MHC	Major histocompatibility complex
Non-IPF Control	Small airway cell cultures generated from patients without a history of IPF
Normal samples	Tissue samples taken from donor lung tissue and used for IHC
Post-COVID fibrosis	Sample group consisting of patients diagnosed with ILD before being infected by COVID-19
SARS-CoV-2, COVID-19	Severe acute respiratory syndrome coronavirus 2
Small airway	Human airways having a diameter of less than 2 mm
TGF-β1	Transforming growth factor beta 1
Treated cultures	Small airway cell cultures treated with TGF-β1 for 48 hours
UIP	Usual interstitial pneumonia
Untreated/baseline cultures	Small airway cell cultures not treated with TGF-β1
