# Supplementary material for: Differential Transcriptomic Signatures of Small Airway Cell Cultures Derived from IPF and COVID-19-Induced Exacerbation of Interstitial Lung Disease
Source: Cells. 2023 Oct 21;12(20):2501. doi: 10.3390/cells12202501 (PMC10605205; doi:10.3390/cells12202501)
Supplement: Supplementary file 1 [file cells-12-02501-s001.zip › cells-2614249-supplementary/Table S2.pdf]

Supplementary Table S2. List of primer sequences used for RT-PCR analysis.

**Target**	**Sequence (5'-3')**
Human ACTB Forward	GGATCAGCAAGCAGGAGTATG
Human ACTB Reverse	AGAAAGGGTGTAACGCAACTAA
Human BMP7 Forward	CCTAACCAAGTGTCCCGATTT
Human BMP7 Reverse	GGAGGCTGAGTGCATACTATT T
Human BMPR1A Forward	AGTGGGTCTGGACTACCTTTA
Human BMPR1A Reverse	GCCCATCCATACTTCTCCATATC
Human BMPR1B Forward	CCTATACACCACAGGGCTTTAC
Human BMPR1B Reverse	CGAGGTCTGGTTTCTTGTCTT
Human FOXM1 Forward	CAGGGTGGTCCGTGTAAATAG
Human FOXM1 Reverse	CTTCTGGCAGTCTCTGGATAAT
