# Supplementary material for: Differential Transcriptomic Signatures of Small Airway Cell Cultures Derived from IPF and COVID-19-Induced Exacerbation of Interstitial Lung Disease
Source: Cells. 2023 Oct 21;12(20):2501. doi: 10.3390/cells12202501 (PMC10605205; doi:10.3390/cells12202501)
Supplement: Supplementary file 1 [file cells-12-02501-s001.zip › cells-2614249-supplementary/Table S3.pdf]

Supplementary Table S3. Top 100 canonical pathway analysis results for the IPF vs. Normal DEG comparison, as calculated by the Ingenuity Pathway Analysis tool (results sorted by -log(p-value)). A positive z-score indicates that the pathway is predicted to be activated, while a negative z-score signifies predicted inhibition. "N/A" is used when not enough data exists to determine the activation state of the pathway.

**Ingenuity Canonical Pathways**	**-log(p-value)**	**Ratio**	**z-score**
Hepatic Fibrosis / Hepatic Stellate Cell Activation	12.4	0.402	N/A
Kinetochore Metaphase Signaling Pathway	8.12	0.414	3.656
Wound Healing Signaling Pathway	8.08	0.329	-5.159
Pulmonary Fibrosis Idiopathic Signaling Pathway	7.75	0.307	-5.511
Axonal Guidance Signaling	7.41	0.277	N/A
Chondroitin Sulfate Biosynthesis (Late Stages)	7.25	0.52	-0.784
Xenobiotic Metabolism CAR Signaling Pathway	7.08	0.34	1.861
Chondroitin Sulfate Biosynthesis	6.86	0.483	-1.134
LPS/IL-1 Mediated Inhibition of RXR Function	6.22	0.307	-1.414
GP6 Signaling Pathway	6.06	0.362	-4.629
Xenobiotic Metabolism PXR Signaling Pathway	5.89	0.323	1.778
Dermatan Sulfate Biosynthesis (Late Stages)	5.88	0.489	-0.626
Dermatan Sulfate Biosynthesis	5.87	0.45	-1.347
Superpathway of Melatonin Degradation	5.83	0.433	0.186
G-Protein Coupled Receptor Signaling	5.79	0.25	-3.618
Breast Cancer Regulation by Stathmin1	5.65	0.256	-2.887
Melatonin Degradation I	5.53	0.435	0.192

Phagosome Formation	5.35	0.247	-4.575
CREB Signaling in Neurons	5.29	0.252	-2.833
Regulation of Cellular Mechanics by Calpain Protease	5.23	0.382	-2.84
STAT3 Pathway	5.23	0.341	-1.257
ID1 Signaling Pathway	5.16	0.308	-1.016
Xenobiotic Metabolism Signaling	5.16	0.285	N/A
Estrogen-mediated S-phase Entry	5.15	0.577	1.291
Nicotine Degradation III	5.08	0.431	0.408
Nicotine Degradation II	4.92	0.409	0.962
Molecular Mechanisms of Cancer	4.83	0.26	N/A
Agranulocyte Adhesion and Diapedesis	4.81	0.3	N/A
RHOGDI Signaling	4.7	0.295	3.452
Glioblastoma Multiforme Signaling	4.57	0.31	-1.265
Actin Cytoskeleton Signaling	4.55	0.287	-3.904
S100 Family Signaling Pathway	4.3	0.236	-3.113
Tumor Microenvironment Pathway	4.29	0.302	-3.81
Signaling by Rho Family GTPases	3.97	0.273	-3.221
ILK Signaling	3.95	0.289	-2.887
Bupropion Degradation	3.9	0.52	0.832
Role Of Osteoclasts In Rheumatoid Arthritis Signaling Pathway	3.9	0.265	-4.756
Serotonin Degradation	3.8	0.366	0.392

Pathogen Induced Cytokine Storm Signaling Pathway	3.79	0.256	-4.412
Cardiac Hypertrophy Signaling (Enhanced)	3.75	0.242	-1.782
Semaphorin Signaling in Neurons	3.64	0.377	N/A
Atherosclerosis Signaling	3.63	0.308	N/A
Semaphorin Neuronal Repulsive Signaling Pathway	3.62	0.3	-0.617
Agrin Interactions at Neuromuscular Junction	3.59	0.362	-1.964
PAK Signaling	3.58	0.316	-2.041
Integrin Signaling	3.55	0.278	-3.501
Neuroinflammation Signaling Pathway	3.51	0.259	-1.016
Osteoarthritis Pathway	3.46	0.271	-1.605
Role of Osteoblasts, Osteoclasts and Chondrocytes in Rheumatoid Arthritis	3.4	0.272	N/A
Estrogen Biosynthesis	3.33	0.4	0.728
Cellular Effects of Sildenafil (Viagra)	3.32	0.293	N/A
Sperm Motility	3.3	0.265	-1.3
Pulmonary Healing Signaling Pathway	3.27	0.276	-2.562
Heparan Sulfate Biosynthesis	3.26	0.33	-0.928
Actin Nucleation by ARP-WASP Complex	3.18	0.323	-1.807
Heparan Sulfate Biosynthesis (Late Stages)	3.16	0.333	-0.577
Cell Cycle: G2/M DNA Damage Checkpoint Regulation	3.16	0.38	-2.357
Germ Cell-Sertoli Cell Junction Signaling	3.15	0.282	N/A
Sertoli Cell-Sertoli Cell Junction Signaling	3.13	0.272	N/A

Endocannabinoid Neuronal Synapse Pathway	3.1	0.289	-1.029
Granulocyte Adhesion and Diapedesis	3.08	0.275	N/A
Thyroid Hormone Metabolism II (via Conjugation and/or Degradation)	3.02	0.4	-0.5
Hepatic Fibrosis Signaling Pathway	3.02	0.241	-4.72
Mitotic Roles of Polo-Like Kinase	2.97	0.343	1.5
Caveolar-mediated Endocytosis Signaling	2.97	0.333	N/A
Neutrophil Extracellular Trap Signaling Pathway	2.86	0.24	2.915
CDX Gastrointestinal Cancer Signaling Pathway	2.86	0.267	0.816
Apelin Liver Signaling Pathway	2.86	0.444	-1.732
Ephrin Receptor Signaling	2.86	0.267	-2.263
Role Of Osteoblasts In Rheumatoid Arthritis Signaling Pathway	2.82	0.258	-3.048
Notch Signaling	2.8	0.395	-0.302
Regulation of Actin-based Motility by Rho	2.77	0.296	-2.132
Aryl Hydrocarbon Receptor Signaling	2.75	0.277	-0.853
Paxillin Signaling	2.73	0.299	-2.683
Transcriptional Regulatory Network in Embryonic Stem Cells	2.69	0.352	N/A
Histidine Degradation VI	2.69	0.429	-0.333
MSP-RON Signaling Pathway	2.69	0.345	N/A
Xenobiotic Metabolism AHR Signaling Pathway	2.64	0.31	0.192
Dopamine Degradation	2.63	0.406	0.277
Airway Pathology in Chronic Obstructive Pulmonary Disease	2.56	0.288	N/A

Adrenomedullin signaling pathway	2.55	0.261	-1.18
Dilated Cardiomyopathy Signaling Pathway	2.5	0.273	0.898
Cyclins and Cell Cycle Regulation	2.47	0.306	1.877
Colorectal Cancer Metastasis Signaling	2.46	0.247	-2.177
Glioma Invasiveness Signaling	2.42	0.315	-1.147
Macrophage Alternative Activation Signaling Pathway	2.41	0.257	-0.277
Gustation Pathway	2.36	0.256	0
HMGB1 Signaling	2.32	0.263	-1.347
Intrinsic Prothrombin Activation Pathway	2.31	0.357	-2.84
Pregnenolone Biosynthesis	2.31	0.407	0
Regulation of eIF4 and p70S6K Signaling	2.31	0.26	-1.155
Gαi Signaling	2.31	0.271	-2.556
Acetone Degradation I (to Methylglyoxal)	2.2	0.349	1.291
Leukocyte Extravasation Signaling	2.18	0.254	-2.53
Reelin Signaling in Neurons	2.17	0.268	-1.89
Sphingosine-1-phosphate Signaling	2.17	0.275	-0.928
Bladder Cancer Signaling	2.14	0.276	-0.535
Role of Tissue Factor in Cancer	2.14	0.276	N/A
Synaptogenesis Signaling Pathway	2.07	0.235	-4.154
Human Embryonic Stem Cell Pluripotency	2.03	0.249	-1.131
