# Supplementary material for: Differential Transcriptomic Signatures of Small Airway Cell Cultures Derived from IPF and COVID-19-Induced Exacerbation of Interstitial Lung Disease
Source: Cells. 2023 Oct 21;12(20):2501. doi: 10.3390/cells12202501 (PMC10605205; doi:10.3390/cells12202501)
Supplement: Supplementary file 1 [file cells-12-02501-s001.zip › cells-2614249-supplementary/Table S4.pdf]

Supplementary Table S4. Top 100 upstream regulator results for the IPF vs. Normal DEG comparison, as calculated by the Ingenuity Pathway Analysis tool (results sorted by p-value of the overlap) A positive z-score indicates that the regulator is predicted to be activated, while a negative z-score signifies predicted inhibition.

**Upstream Regulator**	**Activation z-score**	**p-value of overlap**
ELDR	7.319	1.13E-37
TGFB1	-9.332	8.24E-36
TNF	-6.573	9.75E-31
ERBB2	-1.533	3.07E-28
IL1B	-3.92	3.12E-28
YAP1	-2.129	6.68E-25
CTNNB1	-3.522	2.2E-24
FOXM1	2.633	2.26E-24
CKAP2L	5.642	5.31E-24
ESR2	-0.378	6.9E-24
TP53	-5.282	1.16E-23
TP63	0.126	1.17E-23
PGR	-0.64	3.13E-23
Vegf	1.201	6.2E-23
MAP2K1	-3.49	1.4E-21
AGT	-6.13	1.44E-21
IL6	-4.097	4.07E-21
CDKN1A	-1.773	6.76E-21

CCND1	0.26	1.78E-20
HRAS	-1.046	2.8E-20
CSF2	1.451	3.55E-20
CG	-2.968	1.18E-19
ZBTB17		1.62E-19
STAT3	-1.533	1.99E-19
CEBPB	2.639	2.3E-19
PCLAF	5.261	2.39E-19
PTGER2	2.889	3.29E-19
SMARCA4	-2.976	2.22E-18
MYOD1	-0.12	1.61E-17
ERBB3	-0.463	9.59E-17
SP1	-3.624	9.88E-17
MRTFB	-3.028	1.38E-16
TCF3	-2.978	1.42E-16
AMBRA1	3.647	1.66E-16
NUPR1	-8.034	1.96E-16
NKX2-3	-0.226	2.1E-16
AHR	0.858	2.24E-16
CDKN2A	-5.089	2.84E-16
IFNG	-3.853	3.48E-16

Tgf beta	-5.273	9.47E-16
E2F4	-1.612	1.66E-15
GLI1	-1.302	2.58E-15
KRAS	-0.425	4.49E-15
estrogen receptor	2.611	1.47E-14
TP73	-1.332	2.4E-14
HDAC1	2.609	2.7E-14
FGF2	-3.626	2.75E-14
TWIST1	-2.567	7.84E-14
CASR	1.13	1.24E-13
HGF	1.067	1.55E-13
AREG	1.767	1.58E-13
MYC	3.046	1.94E-13
WWTR1	-0.126	4.97E-13
IGF1	-2.921	5.42E-13
YY1	0.376	6.62E-13
SMAD3	-5.08	7.96E-13
WNT3A	-3.069	8.87E-13
BRD4	-0.735	1.22E-12
RABL6	5.754	1.43E-12
STAT1	-0.733	1.69E-12

NRAS	-0.316	1.71E-12
Interferon alpha	0.174	1.72E-12
SP3	0.252	2.27E-12
RB1	-1.642	2.88E-12
KDM1A	3.467	3.41E-12
PDGF BB	-2.635	4.12E-12
NRG1	-2.541	4.27E-12
TGFB2	-4.838	4.83E-12
let-7	-0.697	6.12E-12
EDN1	-5.168	7.34E-12
MRTFA	-3.174	7.73E-12
AR	-1.507	9E-12
EZH2	0.142	9.2E-12
PDLIM2	0.493	9.21E-12
ESR1	2.595	1.04E-11
MYCN	5.907	1.08E-11
FKBP10	4.802	1.44E-11
KDM3B	-1.89	1.52E-11
ITGB1	-1.1	2.11E-11
F2	-6.066	2.17E-11
IL13	-0.389	2.36E-11

NOTCH3	-3.214	2.47E-11
HNRNPA2B1	-1.982	2.5E-11
PELP1	-1.436	2.65E-11
FOS	-3.835	3.98E-11
ZNF768	4.347	4.72E-11
ETV5	0.148	4.73E-11
MAPK1	-2.687	4.9E-11
HIF1A	-3.832	4.92E-11
MYB	0.072	5.43E-11
EGF	-4.324	6.16E-11
MACROH2A1	1.616	6.21E-11
RARA	1.634	6.44E-11
E2f	2.875	6.9E-11
SOX9	-2.267	8.14E-11
NPM1	-5.295	8.97E-11
RUNX2	-0.91	9.85E-11
ID2	0.053	1.38E-10
CDK4	0.059	1.39E-10
TGFBR1	-0.16	1.43E-10
