# Supplementary material for: Differential Transcriptomic Signatures of Small Airway Cell Cultures Derived from IPF and COVID-19-Induced Exacerbation of Interstitial Lung Disease
Source: Cells. 2023 Oct 21;12(20):2501. doi: 10.3390/cells12202501 (PMC10605205; doi:10.3390/cells12202501)
Supplement: Supplementary file 1 [file cells-12-02501-s001.zip › cells-2614249-supplementary/Table S5.pdf]

Supplementary Table S5. Top 100 gene ontology results for the IPF vs. Normal upregulated DEGs

GO biological process complete	Fold Enrichment	Raw P-value	FDR
mucociliary clearance (GO:0120197)	7.99	2.84E-04	2.17E-02
axonemal dynein complex assembly (GO:0070286)	7.08	1.32E-13	9.03E-11
inner dynein arm assembly (GO:0036159)	6.99	1.94E-06	2.76E-04
outer dynein arm assembly (GO:0036158)	6.75	7.23E-08	1.47E-05
epithelial cilium movement involved in extracellular fluid movement (GO:0003351)	6.52	1.45E-12	7.84E-10
intraciliary retrograde transport (GO:0035721)	6.52	4.58E-05	4.43E-03
axoneme assembly (GO:0035082)	6.44	4.29E-26	7.48E-23
regulation of mitotic cytokinesis (GO:1902412)	6.39	7.19E-04	4.73E-02
kinetochore organization (GO:0051383)	6.39	1.64E-06	2.46E-04
epithelial cilium movement involved in determination of left/right asymmetry (GO:0060287)	6.32	1.31E-04	1.11E-02
intraciliary transport (GO:0042073)	6.22	6.67E-13	3.88E-10
cerebrospinal fluid circulation (GO:0090660)	6.09	6.96E-05	6.42E-03
extracellular transport (GO:0006858)	6.09	5.13E-12	2.59E-09
intraciliary anterograde transport (GO:0035720)	5.91	3.68E-05	3.68E-03
sperm axoneme assembly (GO:0007288)	5.87	1.38E-07	2.48E-05
spindle elongation (GO:0051231)	5.62	5.56E-04	3.94E-02

mitotic spindle assembly checkpoint signaling (GO:0007094)	5.6	1.08E-07	2.07E-05
mitotic spindle checkpoint signaling (GO:0071174)	5.6	1.08E-07	2.04E-05
spindle assembly checkpoint signaling (GO:0071173)	5.6	1.08E-07	2.02E-05
negative regulation of mitotic metaphase/anaphase transition (GO:0045841)	5.54	5.75E-08	1.20E-05
kinetochore assembly (GO:0051382)	5.48	2.88E-04	2.19E-02
motile cilium assembly (GO:0044458)	5.45	2.13E-13	1.28E-10
spindle checkpoint signaling (GO:0031577)	5.42	1.56E-07	2.78E-05
microtubule bundle formation (GO:0001578)	5.36	4.57E-25	7.17E-22
negative regulation of mitotic sister chromatid separation (GO:2000816)	5.33	4.40E-08	9.85E-06
negative regulation of mitotic sister chromatid segregation (GO:0033048)	5.33	4.40E-08	9.71E-06
negative regulation of sister chromatid segregation (GO:0033046)	5.33	4.40E-08	9.57E-06
negative regulation of metaphase/anaphase transition of cell cycle (GO:1902100)	5.22	1.18E-07	2.18E-05
protein localization to chromosome, centromeric region (GO:0071459)	5.12	1.11E-05	1.28E-03
sperm flagellum assembly (GO:0120316)	5.07	1.67E-07	2.91E-05
negative regulation of chromosome segregation (GO:0051985)	5.05	8.81E-08	1.73E-05
negative regulation of chromosome separation (GO:1905819)	5.05	8.81E-08	1.70E-05

negative regulation of mitotic nuclear division (GO:0045839)	4.9	6.48E-08	1.34E-05
regulation of cilium beat frequency (GO:0003356)	4.84	5.73E-04	4.03E-02
centromere complex assembly (GO:0034508)	4.74	2.14E-05	2.25E-03
negative regulation of nuclear division (GO:0051784)	4.38	1.60E-07	2.81E-05
cilium movement (GO:0003341)	4.3	2.77E-22	3.35E-19
cilium organization (GO:0044782)	4.25	2.48E-44	3.89E-40
positive regulation of chromosome separation (GO:1905820)	4.24	1.01E-04	8.93E-03
attachment of spindle microtubules to kinetochore (GO:0008608)	4.22	1.94E-04	1.55E-02
cilium assembly (GO:0060271)	4.14	2.50E-38	1.31E-34
regulation of cilium movement (GO:0003352)	4.04	4.77E-06	5.75E-04
cilium-dependent cell motility (GO:0060285)	3.96	1.53E-15	1.50E-12
cilium or flagellum-dependent cell motility (GO:0001539)	3.96	1.53E-15	1.41E-12
regulation of mitotic sister chromatid segregation (GO:0033047)	3.91	4.34E-07	6.87E-05
cilium movement involved in cell motility (GO:0060294)	3.64	4.50E-12	2.35E-09
chromosome condensation (GO:0030261)	3.61	4.62E-05	4.45E-03
mitotic spindle organization (GO:0007052)	3.58	1.60E-09	4.64E-07
microtubule-based movement (GO:0007018)	3.49	5.15E-32	1.35E-28
sperm motility (GO:0097722)	3.46	1.94E-10	7.23E-08

flagellated sperm motility (GO:0030317)	3.46	1.94E-10	7.06E-08
protein localization to cilium (GO:0061512)	3.44	4.29E-06	5.26E-04
plasma membrane bounded cell projection assembly (GO:0120031)	3.43	1.74E-33	6.80E-30
mitotic sister chromatid segregation (GO:0000070)	3.43	1.63E-10	6.24E-08
mitotic spindle assembly (GO:0090307)	3.4	3.27E-05	3.35E-03
non-motile cilium assembly (GO:1905515)	3.37	1.47E-05	1.61E-03
cell projection assembly (GO:0030031)	3.36	8.18E-33	2.56E-29
microtubule cytoskeleton organization involved in mitosis (GO:1902850)	3.26	3.91E-10	1.33E-07
regulation of microtubule-based movement (GO:0060632)	3.25	2.24E-05	2.34E-03
microtubule-based transport (GO:0099111)	3.19	1.59E-14	1.39E-11
regulation of cilium assembly (GO:1902017)	3.18	1.23E-05	1.38E-03
regulation of mitotic nuclear division (GO:0007088)	3.17	6.34E-09	1.74E-06
regulation of chromosome separation (GO:1905818)	3.13	3.09E-08	7.45E-06
sister chromatid segregation (GO:0000819)	3.02	8.35E-10	2.67E-07
determination of bilateral symmetry (GO:0009855)	2.93	1.89E-08	4.70E-06
determination of left/right symmetry (GO:0007368)	2.93	4.24E-08	9.78E-06
microtubule cytoskeleton organization (GO:0000226)	2.92	3.06E-30	6.86E-27
specification of symmetry (GO:0009799)	2.91	2.07E-08	5.08E-06

mitotic nuclear division (GO:0140014)	2.9	3.19E-10	1.14E-07
microtubule-based process (GO:0007017)	2.89	1.55E-43	1.22E-39
regulation of mitotic sister chromatid separation (GO:0010965)	2.89	3.66E-06	4.63E-04
regulation of metaphase/anaphase transition of cell cycle (GO:1902099)	2.88	5.50E-06	6.48E-04
regulation of mitotic metaphase/anaphase transition (GO:0030071)	2.88	4.91E-06	5.83E-04
mitotic metaphase plate congression (GO:0007080)	2.87	6.52E-04	4.39E-02
regulation of chromosome segregation (GO:0051983)	2.86	8.62E-08	1.71E-05
protein localization to chromosome (GO:0034502)	2.8	1.01E-04	8.92E-03
spindle organization (GO:0007051)	2.8	7.25E-09	1.96E-06
regulation of nuclear division (GO:0051783)	2.77	5.25E-08	1.11E-05
spindle assembly (GO:0051225)	2.76	1.19E-05	1.35E-03
metaphase plate congression (GO:0051310)	2.75	5.18E-04	3.76E-02
centrosome cycle (GO:0007098)	2.74	2.65E-05	2.75E-03
regulation of sister chromatid segregation (GO:0033045)	2.73	4.60E-06	5.59E-04
nuclear chromosome segregation (GO:0098813)	2.69	3.59E-11	1.52E-08
negative regulation of chromosome organization (GO:2001251)	2.67	9.90E-05	8.82E-03
cytoplasmic translation (GO:0002181)	2.65	2.93E-06	3.80E-04
chromosome segregation (GO:0007059)	2.61	1.21E-12	6.78E-10

microtubule organizing center organization (GO:0031023)	2.59	3.08E-05	3.18E-03
smoothened signaling pathway (GO:0007224)	2.54	1.26E-04	1.07E-02
chromosome localization (GO:0050000)	2.53	2.43E-04	1.90E-02
regulation of cytokinesis (GO:0032465)	2.53	1.47E-04	1.20E-02
establishment of chromosome localization (GO:0051303)	2.52	4.71E-04	3.45E-02
organelle assembly (GO:0070925)	2.5	8.13E-30	1.59E-26
protein-containing complex localization (GO:0031503)	2.48	2.07E-06	2.88E-04
meiotic chromosome segregation (GO:0045132)	2.47	1.25E-04	1.06E-02
xenobiotic metabolic process (GO:0006805)	2.42	4.54E-05	4.42E-03
nuclear division (GO:0000280)	2.4	2.38E-11	1.07E-08
transport along microtubule (GO:0010970)	2.38	4.15E-06	5.17E-04
meiotic nuclear division (GO:0140013)	2.38	1.69E-06	2.48E-04
mitotic cell cycle checkpoint signaling (GO:0007093)	2.37	4.33E-05	4.25E-03
nucleosome assembly (GO:0006334)	2.36	4.78E-04	3.49E-02
