# Supplementary material for: Differential Transcriptomic Signatures of Small Airway Cell Cultures Derived from IPF and COVID-19-Induced Exacerbation of Interstitial Lung Disease
Source: Cells. 2023 Oct 21;12(20):2501. doi: 10.3390/cells12202501 (PMC10605205; doi:10.3390/cells12202501)
Supplement: Supplementary file 1 [file cells-12-02501-s001.zip › cells-2614249-supplementary/Table S6.pdf]

**Supplementary Table S6.** Top 100 gene ontology results for the downregulated DEGs in for the IPF vs. Normal comparison.

| GO biological process complete | Fold Enrichment | Raw P-value | FDR |
|---|---|---|---|
| regulation of Rho-dependent protein serine/threonine kinase activity (GO:2000298) | 8.15 | 1.96E-03 | 3.94E-02 |
| norepinephrine uptake (GO:0051620) | 8.15 | 1.96E-03 | 3.94E-02 |
| norepinephrine transport (GO:0015874) | 8.15 | 1.96E-03 | 3.93E-02 |
| positive regulation of synaptic plasticity (GO:0031915) | 7.61 | 3.15E-04 | 9.35E-03 |
| bone trabecula formation (GO:0060346) | 7.34 | 9.85E-04 | 2.31E-02 |
| positive regulation of extracellular matrix disassembly (GO:0090091) | 6.52 | 1.51E-03 | 3.23E-02 |
| peptidyl-lysine hydroxylation (GO:0017185) | 6.52 | 1.51E-03 | 3.23E-02 |
| skeletal myofibril assembly (GO:0014866) | 6.52 | 1.51E-03 | 3.22E-02 |
| response to macrophage colony-stimulating factor (GO:0036005) | 6.52 | 2.45E-04 | 7.50E-03 |
| positive regulation of podosome assembly (GO:0071803) | 6.22 | 7.41E-04 | 1.84E-02 |
| retina vasculature morphogenesis in camera-type eye (GO:0061299) | 6.22 | 7.41E-04 | 1.84E-02 |
| collagen biosynthetic process (GO:0032964) | 5.87 | 2.23E-03 | 4.36E-02 |
| vascular associated smooth muscle cell development (GO:0097084) | 5.71 | 1.08E-03 | 2.48E-02 |
| postsynaptic cytoskeleton organization (GO:0099188) | 5.27 | 1.53E-03 | 3.24E-02 |
| monocyte differentiation (GO:0030224) | 5.15 | 1.77E-04 | 5.81E-03 |
| retina vasculature development in camera-type eye (GO:0061298) | 5.15 | 1.77E-04 | 5.80E-03 |
| regulation of podosome assembly (GO:0071801) | 4.89 | 2.11E-03 | 4.18E-02 |

| | | | |
|---|---|---|---|
| fructose metabolic process (GO:0006000) | 4.89 | 2.11E-03 | 4.17E-02 |
| vitamin D metabolic process (GO:0042359) | 4.89 | 2.11E-03 | 4.17E-02 |
| mesenchymal to epithelial transition (GO:0060231) | 4.89 | 1.02E-03 | 2.38E-02 |
| surfactant homeostasis (GO:0043129) | 4.89 | 1.02E-03 | 2.37E-02 |
| regulation of extracellular matrix disassembly (GO:0010715) | 4.89 | 1.02E-03 | 2.37E-02 |
| neurotransmitter reuptake (GO:0098810) | 4.68 | 1.62E-04 | 5.38E-03 |
| regulation of endothelial cell chemotaxis (GO:2001026) | 4.66 | 3.30E-04 | 9.64E-03 |
| positive regulation of cell migration involved in sprouting angiogenesis (GO:0090050) | 4.66 | 3.30E-04 | 9.62E-03 |
| collagen fibril organization (GO:0030199) | 4.56 | 2.96E-09 | 3.84E-07 |
| cellular response to ATP (GO:0071318) | 4.35 | 1.84E-03 | 3.77E-02 |
| chemical homeostasis within a tissue (GO:0048875) | 4.35 | 1.84E-03 | 3.77E-02 |
| vascular associated smooth muscle cell differentiation (GO:0035886) | 4.35 | 1.84E-03 | 3.76E-02 |
| endodermal cell differentiation (GO:0035987) | 4.22 | 2.49E-06 | 1.57E-04 |
| positive regulation of extracellular matrix organization (GO:1903055) | 4.19 | 1.86E-04 | 6.02E-03 |
| positive regulation of heart rate (GO:0010460) | 4.14 | 3.74E-04 | 1.07E-02 |
| cell adhesion mediated by integrin (GO:0033627) | 4.06 | 1.27E-05 | 6.46E-04 |
| positive regulation of humoral immune response (GO:0002922) | 4 | 1.52E-03 | 3.23E-02 |
| skeletal muscle contraction (GO:0003009) | 3.91 | 3.09E-04 | 9.26E-03 |
| neurotransmitter uptake (GO:0001504) | 3.91 | 3.09E-04 | 9.24E-03 |
| regulation of osteoblast proliferation (GO:0033688) | 3.84 | 6.16E-04 | 1.62E-02 |

| | | | |
|---|---|---|---|
| regulation of extracellular matrix organization (GO:1903053) | 3.84 | 1.46E-06 | 9.97E-05 |
| epiboly involved in wound healing (GO:0090505) | 3.84 | 6.16E-04 | 1.61E-02 |
| wound healing, spreading of cells (GO:0044319) | 3.84 | 6.16E-04 | 1.61E-02 |
| neuromuscular synaptic transmission (GO:0007274) | 3.83 | 1.95E-03 | 3.95E-02 |
| kidney vasculature development (GO:0061440) | 3.83 | 1.95E-03 | 3.94E-02 |
| renal system vasculature development (GO:0061437) | 3.83 | 1.95E-03 | 3.94E-02 |
| endocardial cushion formation (GO:0003272) | 3.83 | 1.95E-03 | 3.93E-02 |
| multicellular organism aging (GO:0010259) | 3.76 | 1.23E-03 | 2.74E-02 |
| steroid catabolic process (GO:0006706) | 3.76 | 1.23E-03 | 2.74E-02 |
| epiboly (GO:0090504) | 3.71 | 7.80E-04 | 1.92E-02 |
| positive regulation of vascular endothelial growth factor production (GO:0010575) | 3.71 | 7.80E-04 | 1.91E-02 |
| cell-substrate junction assembly (GO:0007044) | 3.67 | 1.02E-04 | 3.67E-03 |
| cell surface receptor signaling pathway involved in heart development (GO:0061311) | 3.67 | 2.46E-03 | 4.72E-02 |
| aortic valve morphogenesis (GO:0003180) | 3.67 | 4.95E-04 | 1.35E-02 |
| extracellular matrix assembly (GO:0085029) | 3.63 | 3.14E-04 | 9.35E-03 |
| protein hydroxylation (GO:0018126) | 3.62 | 1.55E-03 | 3.27E-02 |
| positive regulation of macrophage migration (GO:1905523) | 3.62 | 1.55E-03 | 3.27E-02 |
| developmental induction (GO:0031128) | 3.62 | 1.55E-03 | 3.26E-02 |
| semi-lunar valve development (GO:1905314) | 3.58 | 1.27E-04 | 4.43E-03 |
| regulation of calcium ion-dependent exocytosis (GO:0017158) | 3.58 | 1.27E-04 | 4.42E-03 |

| | | | |
|---|---|---|---|
| cell-substrate junction organization (GO:0150115) | 3.58 | 1.27E-04 | 4.41E-03 |
| apoptotic cell clearance (GO:0043277) | 3.56 | 8.13E-05 | 3.04E-03 |
| collagen metabolic process (GO:0032963) | 3.53 | 4.51E-06 | 2.60E-04 |
| cytokine production (GO:0001816) | 3.49 | 1.93E-03 | 3.92E-02 |
| mesenchymal cell proliferation (GO:0010463) | 3.49 | 1.93E-03 | 3.91E-02 |
| integrin-mediated signaling pathway (GO:0007229) | 3.49 | 6.15E-09 | 7.36E-07 |
| morphogenesis of an epithelial sheet (GO:0002011) | 3.45 | 4.10E-05 | 1.77E-03 |
| endoderm formation (GO:0001706) | 3.44 | 2.62E-05 | 1.22E-03 |
| regulation of dopamine secretion (GO:0014059) | 3.44 | 4.85E-04 | 1.33E-02 |
| aortic valve development (GO:0003176) | 3.44 | 4.85E-04 | 1.33E-02 |
| musculoskeletal movement (GO:0050881) | 3.41 | 1.95E-04 | 6.25E-03 |
| multicellular organismal movement (GO:0050879) | 3.41 | 1.95E-04 | 6.24E-03 |
| branching involved in ureteric bud morphogenesis (GO:0001658) | 3.4 | 1.24E-04 | 4.33E-03 |
| heart valve morphogenesis (GO:0003179) | 3.38 | 3.22E-05 | 1.44E-03 |
| regulation of astrocyte differentiation (GO:0048710) | 3.37 | 2.38E-03 | 4.61E-02 |
| substrate adhesion-dependent cell spreading (GO:0034446) | 3.37 | 2.06E-05 | 9.88E-04 |
| vasoconstriction (GO:0042310) | 3.35 | 9.44E-04 | 2.23E-02 |
| regulation of cell migration involved in sprouting angiogenesis (GO:0090049) | 3.35 | 5.96E-04 | 1.58E-02 |
| semaphorin-plexin signaling pathway (GO:0071526) | 3.26 | 4.61E-04 | 1.28E-02 |
| positive regulation of axon extension (GO:0045773) | 3.26 | 7.29E-04 | 1.83E-02 |
| heterotypic cell-cell adhesion (GO:0034113) | 3.26 | 1.15E-03 | 2.61E-02 |

| | | | |
|---|---|---|---|
| regulation of transforming growth factor beta production (GO:0071634) | 3.26 | 7.29E-04 | 1.83E-02 |
| ureteric bud morphogenesis (GO:0060675) | 3.26 | 4.81E-05 | 2.00E-03 |
| positive regulation of protein tyrosine kinase activity (GO:0061098) | 3.26 | 3.07E-05 | 1.38E-03 |
| metanephric nephron development (GO:0072210) | 3.26 | 1.83E-03 | 3.78E-02 |
| response to ATP (GO:0033198) | 3.26 | 1.83E-03 | 3.78E-02 |
| regulation of axon extension involved in axon guidance (GO:0048841) | 3.26 | 1.83E-03 | 3.77E-02 |
| regulation of vascular endothelial growth factor production (GO:0010574) | 3.26 | 1.83E-03 | 3.77E-02 |
| postsynapse organization (GO:0099173) | 3.22 | 4.36E-07 | 3.47E-05 |
| mesonephric tubule morphogenesis (GO:0072171) | 3.2 | 5.84E-05 | 2.33E-03 |
| platelet aggregation (GO:0070527) | 3.18 | 5.60E-04 | 1.50E-02 |
| positive regulation of blood circulation (GO:1903524) | 3.18 | 8.84E-04 | 2.12E-02 |
| heart valve development (GO:0003170) | 3.16 | 2.88E-05 | 1.32E-03 |
| leukocyte cell-cell adhesion (GO:0007159) | 3.14 | 1.11E-04 | 3.91E-03 |
| positive regulation of signaling receptor activity (GO:2000273) | 3.12 | 4.29E-04 | 1.21E-02 |
| negative regulation of hemostasis (GO:1900047) | 3.12 | 4.29E-04 | 1.20E-02 |
| modulation of excitatory postsynaptic potential (GO:0098815) | 3.11 | 6.76E-04 | 1.73E-02 |
| positive regulation of endothelial cell migration (GO:0010595) | 3.11 | 1.57E-07 | 1.39E-05 |
| smooth muscle contraction (GO:0006939) | 3.1 | 8.49E-05 | 3.15E-03 |
| smooth muscle cell differentiation (GO:0051145) | 3.09 | 1.69E-03 | 3.52E-02 |
| endothelial cell migration (GO:0043542) | 3.08 | 1.71E-05 | 8.36E-04 |

| | | | |
|---|---|---|---|
| positive regulation of blood vessel endothelial cell migration (GO:0043536) | 3.08 | 2.09E-04 | 6.59E-03 |
| artery morphogenesis (GO:0048844) | 3.07 | 4.16E-05 | 1.79E-03 |
