# Supplementary material for: Differential Transcriptomic Signatures of Small Airway Cell Cultures Derived from IPF and COVID-19-Induced Exacerbation of Interstitial Lung Disease
Source: Cells. 2023 Oct 21;12(20):2501. doi: 10.3390/cells12202501 (PMC10605205; doi:10.3390/cells12202501)
Supplement: Supplementary file 1 [file cells-12-02501-s001.zip › cells-2614249-supplementary/Table S7.pdf]

Supplementary Table S7. Top 100 canonical pathway analysis results for the COVID vs. Normal DEG comparison, as calculated by the Ingenuity Pathway Analysis tool (results sorted by -log(p-value)). A positive z-score indicates that the pathway is predicted to be activated, while a negative z-score signifies predicted inhibition. “N/A” is used when not enough data exists to determine the activation state of the pathway.

**Ingenuity Canonical Pathways**	**-log(p-value)**	**Ratio**	**z-score**
Role of Hypercytokinemia/hyperchemokinemia in the Pathogenesis of Influenza	8.56	0.291	-3.4
Interferon Signaling	5.94	0.361	-2.887
CREB Signaling in Neurons	4.98	0.125	-0.12
Pathogen Induced Cytokine Storm Signaling Pathway	4.58	0.137	-0.14
LPS/IL-1 Mediated Inhibition of RXR Function	4.36	0.15	-1
S100 Family Signaling Pathway	4.32	0.115	-2.014
G-Protein Coupled Receptor Signaling	3.98	0.115	-0.333
Phagosome Formation	3.71	0.114	-0.563
Eicosanoid Signaling	3.7	0.214	1.342
Breast Cancer Regulation by Stathmin1	3.58	0.116	-0.855
Neurovascular Coupling Signaling Pathway	3.46	0.142	1.061
Melatonin Degradation I	3.19	0.21	0.832
Multiple Sclerosis Signaling Pathway	3.15	0.14	0.898
Cell Cycle Control of Chromosomal Replication	3.07	0.214	-3.464
Coagulation System	3.03	0.257	-1.667
Macrophage Classical Activation Signaling Pathway	2.97	0.143	0.577
Chondroitin Sulfate Biosynthesis (Late Stages)	2.96	0.22	-0.302

Superpathway of Melatonin Degradation	2.86	0.194	0.832
Synaptic Long Term Depression	2.67	0.136	0.626
Estrogen-mediated S-phase Entry	2.59	0.269	-2.646
Regulation of Cellular Mechanics by Calpain Protease	2.57	0.169	2.449
Salvage Pathways of Pyrimidine Deoxyribonucleotides	2.52	0.444	-2
Role Of Osteoblasts In Rheumatoid Arthritis Signaling Pathway	2.49	0.127	1.095
Chondroitin Sulfate Biosynthesis	2.42	0.19	-0.302
Oxytocin In Spinal Neurons Signaling Pathway	2.4	0.229	1.414
Dermatan Sulfate Biosynthesis	2.3	0.183	-0.302
VDR/RXR Activation	2.25	0.167	-0.632
Gustation Pathway	2.22	0.128	-2.041
Pyrimidine Deoxyribonucleotides De Novo Biosynthesis I	2.21	0.261	-2.449
IL-10 Signaling	2.2	0.136	-1.964
Role of BRCA1 in DNA Damage Response	2.16	0.163	-1.414
Role of Pattern Recognition Receptors in Recognition of Bacteria and Viruses	2.14	0.135	-1.897
Dermatan Sulfate Biosynthesis (Late Stages)	2.1	0.191	-1
Synaptogenesis Signaling Pathway	2.05	0.114	-0.354
Thyroid Hormone Metabolism II (via Conjugation and/or Degradation)	2.03	0.2	0.707
Bupropion Degradation	2.02	0.24	0
Neuroinflammation Signaling Pathway	2	0.114	-0.365
Xenobiotic Metabolism CAR Signaling Pathway	2	0.126	-0.816

Corticotropin Releasing Hormone Signaling	1.96	0.132	0.688
LXR/RXR Activation	1.94	0.138	1.5
Factors Promoting Cardiogenesis in Vertebrates	1.93	0.131	-0.688
Retinoate Biosynthesis I	1.91	0.19	-0.816
Hepatic Fibrosis Signaling Pathway	1.87	0.106	-0.493
Regulation Of The Epithelial Mesenchymal Transition In Development Pathway	1.86	0.149	0.632
PD-1, PD-L1 cancer immunotherapy pathway	1.82	0.14	-0.577
White Adipose Tissue Browning Pathway	1.78	0.13	-0.243
MSP-RON Signaling In Macrophages Pathway	1.75	0.134	-1.5
Estrogen Biosynthesis	1.73	0.178	0
CDX Gastrointestinal Cancer Signaling Pathway	1.72	0.119	0.408
Complement System	1.71	0.189	-1
Kinetochore Metaphase Signaling Pathway	1.69	0.135	-1.155
PXR/RXR Activation	1.62	0.154	-0.632
Activation of IRF by Cytosolic Pattern Recognition Receptors	1.62	0.154	-1.265
Nicotine Degradation II	1.58	0.152	1.265
Glutamate Receptor Signaling	1.58	0.152	-0.447
The Visual Cycle	1.58	0.217	-1.342
Gαs Signaling	1.57	0.128	0
Mitotic Roles of Polo-Like Kinase	1.54	0.149	-2.646
Nicotine Degradation III	1.53	0.155	1

Endocannabinoid Neuronal Synapse Pathway	1.48	0.121	-1.069
Xenobiotic Metabolism PXR Signaling Pathway	1.47	0.115	-1.279
Phospholipases	1.46	0.145	1.134
cAMP-mediated signaling	1.45	0.11	0.408
SPINK1 Pancreatic Cancer Pathway	1.44	0.15	-1
Intrinsic Prothrombin Activation Pathway	1.43	0.167	0.378
HOTAIR Regulatory Pathway	1.4	0.117	-1.698
Serotonin Degradation	1.39	0.141	0.632
Acute Phase Response Signaling	1.38	0.114	0.832
Neuropathic Pain Signaling In Dorsal Horn Neurons	1.38	0.129	-0.832
Heparan Sulfate Biosynthesis (Late Stages)	1.38	0.136	-0.905
Th1 Pathway	1.37	0.123	0
Fatty Acid β-oxidation I	1.34	0.171	1
Cardiac Hypertrophy Signaling (Enhanced)	1.33	0.0959	0
Adrenomedullin signaling pathway	1.32	0.111	0.447
Endocannabinoid Cancer Inhibition Pathway	1.27	0.116	1.069
Macrophage Alternative Activation Signaling Pathway	1.26	0.109	0.426
Cyclins and Cell Cycle Regulation	1.25	0.129	-1.508
GP6 Signaling Pathway	1.24	0.118	0.258
Glutathione-mediated Detoxification	1.24	0.162	-2.236
Semaphorin Neuronal Repulsive Signaling Pathway	1.21	0.113	0

Endothelin-1 Signaling	1.2	0.108	-0.5
Salvage Pathways of Pyrimidine Ribonucleotides	1.2	0.124	-2.887
GPCR-Mediated Nutrient Sensing in Enteroendocrine Cells	1.2	0.119	-2.111
Gαi Signaling	1.19	0.114	0.302
Wound Healing Signaling Pathway	1.17	0.103	0.784
Role Of Chondrocytes In Rheumatoid Arthritis Signaling Pathway	1.17	0.113	-1
Heparan Sulfate Biosynthesis	1.16	0.125	-0.905
Role of WNT/GSK-3β Signaling in the Pathogenesis of Influenza	1.16	0.128	0
Role of MAPK Signaling in Inhibiting the Pathogenesis of Influenza	1.13	0.127	0
Oxytocin In Brain Signaling Pathway	1.11	0.106	-0.218
Pulmonary Healing Signaling Pathway	1.11	0.106	0.655
Sperm Motility	1.09	0.101	1.069
Crosstalk between Dendritic Cells and Natural Killer Cells	1.08	0.121	0.707
STAT3 Pathway	1.06	0.111	0.333
Dopamine Degradation	1.04	0.156	0.447
Differential Regulation of Cytokine Production in Intestinal Epithelial Cells by IL-17A and IL-17F	1.04	0.174	1
Glioma Invasiveness Signaling	1	0.123	0.707
Prolactin Signaling	0.983	0.116	-0.447
Acetone Degradation I (to Methylglyoxal)	0.983	0.14	0
TREM1 Signaling	0.893	0.117	1
