# Supplementary material for: Differential Transcriptomic Signatures of Small Airway Cell Cultures Derived from IPF and COVID-19-Induced Exacerbation of Interstitial Lung Disease
Source: Cells. 2023 Oct 21;12(20):2501. doi: 10.3390/cells12202501 (PMC10605205; doi:10.3390/cells12202501)
Supplement: Supplementary file 1 [file cells-12-02501-s001.zip › cells-2614249-supplementary/Table S8.pdf]

Supplementary Table S8. Top 100 upstream regulator results for the COVID vs. Normal DEG comparison, as calculated by the Ingenuity Pathway Analysis tool (results sorted by p-value of the overlap) A positive z-score indicates that the regulator is predicted to be activated, while a negative z-score signifies predicted inhibition.

Upstream Regulator	Activation z-score	p-value of overlap
IFNL1	-6.85	3.97E-38
STAG2	5.73	5.45E-30
NONO	-6.955	5.65E-30
Interferon alpha	-6.262	1.08E-25
TREX1	5.975	2.85E-24
IFNA2	-6.927	9.48E-23
IRGM	5.513	1.92E-22
RNASEH2B	6.301	8.28E-22
PRL	-4.997	1.49E-21
Irgm1	6.293	1.83E-21
STAT1	-4.847	2.26E-21
MAPK1	4.101	9.53E-21
IFNG	-4.378	2.05E-20
NKX2-3	4.752	3.58E-20
PGR	2.65	2.21E-18
IRF7	-6.28	3.38E-18
CNOT7	2.621	7.68E-18
RNY3	-4.359	2.91E-16

TRIM24	5.032	3.38E-16
IFN Beta	-4.999	3.71E-16
IFNL4	-3.568	1.6E-15
FOXC1	-4.148	3.63E-15
RC3H1	4.6	1.08E-14
IFNB1	-4.878	1.98E-14
TASL	-3.479	2.71E-14
EIF2AK2	-4.061	1.05E-13
IRF1	-5.241	1.8E-13
SLC15A4	-4.357	2.15E-13
STAT2	-3.513	2.18E-13
STING1	-4.517	2.48E-13
TNF	-2.263	3.89E-13
IFNA1/IFNA13	-4.177	5.1E-13
IL6	0.476	6.13E-13
IL1RN	4.845	6.59E-13
Ttc39aos1	4.951	8.01E-13
STAT3	1.987	8.59E-13
PNPT1	4.583	1.05E-12
CEBPB	-3.359	1.51E-12
Ifnar	-4.744	1.55E-12

CGAS	-3.716	1.79E-12
RARA	-1.264	2.29E-12
IRF3	-5.297	2.78E-12
KDM1A	-3.621	2.81E-12
Eldr	-4.762	2.94E-12
miR-182-5p (and other miRNAs w/seed UUGGCAA)	3.396	9.58E-12
IRF5	-4.536	1.2E-11
SENP3	-4.472	1.47E-11
IFNAR2	-2.646	1.47E-11
IFNAR1	-3.028	1.78E-11
ACKR2	4.472	6E-11
CG	0.962	6.61E-11
SMARCA4	0.211	8.15E-11
USP8	4.835	8.32E-11
MAVS	-4.848	1.12E-10
ESR2	0.519	1.55E-10
ZBTB10	-4.275	2.01E-10
NGEF	0.784	2.3E-10
FOXM1	-2.065	2.78E-10
TGFB1	-1.4	6.12E-10
DDX58	-4.226	7.16E-10

Vegf	-2.555	9.04E-10
AGT	-1.395	1.83E-09
TLR3	-0.68	1.84E-09
JAK	-2	2.97E-09
Ifn	-3.237	3.12E-09
VDR	-2.319	5.4E-09
TGM2	-2.241	5.68E-09
NR1H3	0.696	6.5E-09
mir-96	-4.101	6.75E-09
CSF2	-3.226	8.51E-09
Histone h4		1.01E-08
TERT	-3.489	1.45E-08
CCND1	-3.271	1.69E-08
SPI1	-2.8	1.79E-08
Immunoglobulin	-0.408	1.83E-08
PTGER2	-3.283	2.01E-08
KAT6A	-2.584	2.28E-08
E2f	-3.711	2.31E-08
PML	-3.816	0.000000029
HDAC1	1.658	2.99E-08
mir-183	-4.231	3.36E-08

SP1	-1.375	3.72E-08
DUSP1	1.333	4.32E-08
IRF9	-3.256	4.62E-08
RNASEH2A		5.93E-08
TBX2	-3.714	7.01E-08
IRF8	-0.196	7.08E-08
OSM	0.041	8.85E-08
CTNNB1	-1.143	0.000000103
TCF3	2.366	0.000000115
SOCS1	2.784	0.000000122
LIF	0.145	0.000000135
WWTR1	1.05	0.000000144
CLEC12A	-2.425	0.00000015
CITED2	3.243	0.000000153
CASR	-2.79	0.000000161
TLR7	-2.819	0.000000162
SOX9	0.384	0.000000179
IFNA14	-2.581	0.00000021
G protein alpha i	-3.897	0.000000271
