# Supplementary material for: Differential Transcriptomic Signatures of Small Airway Cell Cultures Derived from IPF and COVID-19-Induced Exacerbation of Interstitial Lung Disease
Source: Cells. 2023 Oct 21;12(20):2501. doi: 10.3390/cells12202501 (PMC10605205; doi:10.3390/cells12202501)
Supplement: Supplementary file 1 [file cells-12-02501-s001.zip › cells-2614249-supplementary/Table S9.pdf]

Supplementary Table S9. Top 100 gene ontology results for the upregulated DEGs in for the COVID vs. Normal comparison.

GO biological process complete	Fold Enrichment	Raw P-value	FDR
positive regulation of high-density lipoprotein particle clearance (GO:0010983)	33.86	4.40E-04	2.85E-02
regulation of antigen processing and presentation of peptide antigen via MHC class II (GO:0002586)	25.4	7.54E-04	4.30E-02
regulation of high-density lipoprotein particle clearance (GO:0010982)	25.4	7.54E-04	4.28E-02
calcium ion-regulated exocytosis of neurotransmitter (GO:0048791)	16.93	1.26E-06	4.72E-04
negative regulation of glial cell apoptotic process (GO:0034351)	15.05	3.90E-04	2.63E-02
peptide antigen assembly with MHC class II protein complex (GO:0002503)	14.82	2.54E-06	7.80E-04
MHC class II protein complex assembly (GO:0002399)	14.82	2.54E-06	7.65E-04
surfactant homeostasis (GO:0043129)	14.82	2.54E-06	7.50E-04
prostate gland growth (GO:0060736)	14.11	8.87E-05	8.43E-03
hyperosmotic salinity response (GO:0042538)	13.55	5.33E-04	3.36E-02
chemical homeostasis within a tissue (GO:0048875)	13.17	4.73E-06	1.03E-03
negative regulation of synapse organization (GO:1905809)	12.31	7.11E-04	4.16E-02
regulation of glial cell apoptotic process (GO:0034350)	12.31	7.11E-04	4.14E-02
peptide antigen assembly with MHC protein complex (GO:0002501)	11.85	8.31E-06	1.53E-03
MHC protein complex assembly (GO:0002396)	11.85	8.31E-06	1.52E-03
neuron cell-cell adhesion (GO:0007158)	10.58	2.65E-04	2.00E-02
nitric oxide mediated signal transduction (GO:0007263)	9.68	9.61E-05	9.03E-03

antigen processing and presentation of exogenous peptide antigen via MHC class II (GO:0019886)	9.03	1.00E-05	1.74E-03
antigen processing and presentation of peptide or polysaccharide antigen via MHC class II (GO:0002504)	8.96	2.92E-06	8.04E-04
antigen processing and presentation of peptide antigen via MHC class II (GO:0002495)	8.47	1.50E-05	2.39E-03
regulation of antigen processing and presentation (GO:0002577)	8.06	7.62E-04	4.30E-02
regulation of urine volume (GO:0035809)	7.7	9.13E-04	4.90E-02
mesenchymal cell proliferation (GO:0010463)	7.26	3.68E-04	2.53E-02
antigen processing and presentation of exogenous peptide antigen (GO:0002478)	6.77	6.00E-05	6.53E-03
negative regulation of blood coagulation (GO:0030195)	6.63	2.43E-05	3.34E-03
long-term memory (GO:0007616)	6.58	2.03E-04	1.62E-02
negative regulation of hemostasis (GO:1900047)	6.48	2.82E-05	3.74E-03
regulation of cholesterol efflux (GO:0010874)	6.41	2.35E-04	1.81E-02
negative regulation of coagulation (GO:0050819)	6.1	4.33E-05	4.99E-03
animal organ formation (GO:0048645)	5.93	3.58E-04	2.50E-02
hydrogen peroxide metabolic process (GO:0042743)	5.93	3.58E-04	2.49E-02
vasodilation (GO:0042311)	5.64	1.85E-04	1.50E-02
antigen processing and presentation of exogenous antigen (GO:0019884)	5.53	2.09E-04	1.67E-02
secondary metabolic process (GO:0019748)	5.42	2.37E-04	1.81E-02
modulation of excitatory postsynaptic potential (GO:0098815)	5.39	5.97E-04	3.62E-02
regulation of blood coagulation (GO:0030193)	5.32	1.94E-05	2.93E-03

calcium-ion regulated exocytosis (GO:0017156)	5.27	6.73E-04	3.99E-02
synaptic vesicle exocytosis (GO:0016079)	5.25	1.20E-04	1.07E-02
regulation of morphogenesis of a branching structure (GO:0060688)	5.21	3.01E-04	2.20E-02
regulation of hemostasis (GO:1900046)	5.17	2.45E-05	3.35E-03
immunoglobulin production involved in immunoglobulin-mediated immune response (GO:0002381)	5.15	7.57E-04	4.29E-02
negative regulation of smooth muscle cell proliferation (GO:0048662)	5.11	3.38E-04	2.41E-02
regulation of systemic arterial blood pressure mediated by a chemical signal (GO:0003044)	5.04	8.49E-04	4.70E-02
regulation of neurotransmitter secretion (GO:0046928)	5.04	2.57E-06	7.47E-04
extracellular matrix disassembly (GO:0022617)	5.04	8.49E-04	4.69E-02
regulation of coagulation (GO:0050818)	4.97	3.44E-05	4.25E-03
renal system process (GO:0003014)	4.96	2.71E-07	1.47E-04
antigen processing and presentation of peptide antigen (GO:0048002)	4.92	1.89E-04	1.53E-02
negative regulation of wound healing (GO:0061045)	4.91	8.51E-05	8.24E-03
regulation of synaptic vesicle exocytosis (GO:2000300)	4.84	2.11E-04	1.67E-02
vascular transport (GO:0010232)	4.73	2.39E-05	3.34E-03
transport across blood-brain barrier (GO:0150104)	4.73	2.39E-05	3.31E-03
positive regulation of lipid transport (GO:0032370)	4.57	3.24E-05	4.17E-03
acute inflammatory response (GO:0002526)	4.54	7.16E-05	7.15E-03
regulation of smooth muscle contraction (GO:0006940)	4.44	7.85E-04	4.38E-02
regulation of neurotransmitter transport (GO:0051588)	4.39	1.09E-05	1.84E-03

antigen processing and presentation (GO:0019882)	4.23	6.32E-05	6.74E-03
kidney epithelium development (GO:0072073)	4.2	2.19E-06	7.00E-04
xenobiotic metabolic process (GO:0006805)	4.05	2.46E-05	3.33E-03
synaptic vesicle cycle (GO:0099504)	4.03	1.35E-05	2.20E-03
blood vessel diameter maintenance (GO:0097746)	4	4.03E-06	9.72E-04
regulation of tube diameter (GO:0035296)	4	4.03E-06	9.58E-04
negative regulation of response to wounding (GO:1903035)	3.98	3.96E-04	2.66E-02
neurotransmitter secretion (GO:0007269)	3.98	3.96E-04	2.64E-02
signal release from synapse (GO:0099643)	3.98	3.96E-04	2.63E-02
regulation of tube size (GO:0035150)	3.97	4.39E-06	9.69E-04
positive regulation of lipid localization (GO:1905954)	3.97	5.83E-05	6.40E-03
vascular process in circulatory system (GO:0003018)	3.93	6.80E-10	1.18E-06
artery development (GO:0060840)	3.92	2.34E-04	1.80E-02
nephron epithelium development (GO:0072009)	3.83	1.49E-04	1.27E-02
vesicle-mediated transport in synapse (GO:0099003)	3.76	2.82E-05	3.72E-03
regulation of systemic arterial blood pressure (GO:0003073)	3.72	3.50E-04	2.48E-02
neutrophil migration (GO:1990266)	3.68	6.99E-04	4.12E-02
regulation of lipid transport (GO:0032368)	3.65	3.84E-05	4.57E-03
regulation of wound healing (GO:0061041)	3.65	7.05E-05	7.23E-03
regulation of regulated secretory pathway (GO:1903305)	3.64	2.26E-05	3.25E-03
regulation of synapse assembly (GO:0051963)	3.62	4.40E-04	2.86E-02

cellular hormone metabolic process (GO:0034754)	3.59	8.19E-05	8.03E-03
cellular response to xenobiotic stimulus (GO:0071466)	3.52	6.18E-06	1.24E-03
nephron development (GO:0072006)	3.51	1.02E-04	9.53E-03
branching morphogenesis of an epithelial tube (GO:0048754)	3.46	1.18E-04	1.06E-02
digestion (GO:0007586)	3.45	6.34E-04	3.78E-02
regulation of lipid localization (GO:1905952)	3.45	2.38E-05	3.36E-03
neurotransmitter transport (GO:0006836)	3.44	1.26E-04	1.11E-02
hormone metabolic process (GO:0042445)	3.4	3.26E-06	8.39E-04
leukocyte chemotaxis (GO:0030595)	3.39	8.51E-05	8.28E-03
regulation of ossification (GO:0030278)	3.33	4.87E-04	3.09E-02
memory (GO:0007613)	3.33	4.87E-04	3.08E-02
morphogenesis of a branching structure (GO:0001763)	3.29	4.10E-05	4.80E-03
regulation of neurotransmitter levels (GO:0001505)	3.19	8.21E-06	1.53E-03
regulation of synapse structure or activity (GO:0050803)	3.14	1.68E-05	2.64E-03
regulation of smooth muscle cell proliferation (GO:0048660)	3.1	5.42E-04	3.40E-02
morphogenesis of a branching epithelium (GO:0061138)	3.08	2.24E-04	1.74E-02
hemostasis (GO:0007599)	3.04	1.58E-04	1.32E-02
extracellular matrix organization (GO:0030198)	3.02	2.79E-06	7.82E-04
extracellular structure organization (GO:0043062)	3.01	2.96E-06	8.01E-04
regulation of blood pressure (GO:0008217)	3	1.18E-04	1.06E-02
external encapsulating structure organization (GO:0045229)	2.99	3.33E-06	8.42E-04

homophilic cell adhesion via plasma membrane adhesion molecules (GO:0007156)	2.99	3.01E-04	2.20E-02
regulation of body fluid levels (GO:0050878)	2.98	9.96E-08	6.25E-05
